# Transport of perfluoroalkyl substances across human induced pluripotent stem cell-derived intestinal epithelial cells in comparison with primary human intestinal epithelial cells and Caco-2 cells

**DOI:** 10.1007/s00204-024-03851-x

**Published:** 2024-08-31

**Authors:** Aafke W. F. Janssen, Loes P. M. Duivenvoorde, Karsten Beekmann, Nicole Pinckaers, Bart van der Hee, Annelies Noorlander, Liz L. Leenders, Jochem Louisse, Meike van der Zande

**Affiliations:** 1 Wageningen Food Safety Research (WFSR), Part of Wageningen University and Research, Akkermaalsbos 2, 6708 WB Wageningen, The Netherlands; 2 Animal Sciences Group, Wageningen University, De Elst 1, 6708 WD Wageningen, The Netherlands; 3 Present Address: European Food Safety Authority (EFSA), Parma, Italy

**Keywords:** PFAS, Toxicology, Stem cells, Gastrointestinal tract, Transport

## Abstract

Humans can be exposed to per- and polyfluoroalkyl substances (PFASs) via many exposure routes, including diet, which may lead to several adverse health effects. So far, little is known about PFAS transport across the human intestinal barrier. In the current study, we aimed to assess the transport of 5 PFASs (PFOS, PFOA, PFNA, PFHxS and HFPO-DA) in a human induced pluripotent stem cell (hiPSC)-derived intestinal epithelial cell (IEC) model. This model was extensively characterized and compared with the widely applied human colonic adenocarcinoma cell line Caco-2 and a human primary IEC-based model, described to most closely resemble in vivo tissue. The hiPSC-derived IEC layers demonstrated polarized monolayers with tight junctions and a mucus layer. The monolayers consisted of enterocytes, stem cells, goblet cells, enteroendocrine cells, and Paneth cells that are also present in native tissue. Transcriptomics analysis revealed distinct differences in gene expression profiles, where the hiPSC-derived IECs showed the highest expression of intestinal tissue-specific genes relative to the primary IEC-based model and the Caco-2 cells clustered closer to the primary IEC-based model than the hiPSC-derived IECs. The order of PFAS transport was largely similar between the models and the apparent permeability (*P*_app_) values of PFAS in apical to basolateral direction in the hiPSC-derived IEC model were in the following order: PFHxS > PFOA > HFPO-DA > PFNA > PFOS. In conclusion, the hiPSC-derived IEC model highly resembles human intestinal physiology and is therefore a promising novel in vitro model to study transport of chemicals across the intestinal barrier for risk assessment of chemicals.

**Supplementary Information:**

The online version contains supplementary material available at 10.1007/s00204-024-03851-x.

## Introduction

Per- and polyfluoroalkyl substances (PFASs) are a group of more than 4700 man-made organic compounds that are used in a wide variety of industrial sectors and consumer products (OECD 2018; Gluge et al. 2020). PFASs consist of a partly or fully fluorinated carbon chain with different chain lengths and attached chemical groups, giving them the desired properties. Due to the strong fluorine–carbon bond, PFASs have an extremely high thermal stability, which, in combination with their hydrophobic and oleophobic properties, renders them desirable for the use in many applications, including firefighting foams, food contact materials, textiles, household products, construction materials and electronics (Gluge et al. 2020; Panieri et al. 2022). However, due to these properties, PFASs are also highly persistent causing them to accumulate in the environment and pose health concerns to all living organisms (Fenton et al. 2021). To that end, perfluorooctane sulfonate (PFOS) was identified as a persistent organic pollutant and included in the Stockholm Convention in 2009, followed by perfluorooctanoic acid (PFOA) in 2019 and by perfluorohexane sulfonate (PFHxS) in 2022, to restrict their production and use (EU 2019, 2020; UNEP 2020, 2022).

PFASs are omnipresent and can be found in the environment including soil, surface water and groundwater, but are also detected in living organisms such as plants, wildlife and humans (Sedlak et al. 2017; Jian et al. 2018; Brusseau et al. 2020; Death et al. 2021; Panieri et al. 2022). Humans can be exposed to PFASs via several routes including ingestion of contaminated food and drinking water, inhalation of air and dust, and dermal absorption (Jian et al. 2018; Kotlarz et al. 2020; Poothong et al. 2020), of which oral exposure is shown to be the predominant route (Vestergren and Cousins 2009; Haug et al. 2011; Poothong et al. 2020). Once ingested, PFASs are absorbed into the bloodstream, where they primarily bind to albumin and are distributed throughout the body with a high affinity for tissues containing high protein levels, mainly blood, liver and kidneys (Pizzurro et al. 2019; ATSDR 2021). PFASs have highly variable serum elimination half-lives ranging from several days for short-chain PFASs to several years for longer-chain PFASs (Olsen et al. 2007; Li et al. 2018; Xu et al. 2020).

Epidemiological studies have demonstrated associations between human exposure to PFASs and hepatoxicity, immunotoxicity, developmental toxicity, elevated cholesterol levels and decreased thyroid hormone levels (EFSA CONTAM Panel 2018, 2020). Although several of these effects have been corroborated by animals studies, there are also inconsistencies. The same holds true for the bioavailability data of PFASs. While most in vivo data show that PFOS and PFOA are readily absorbed in rodents and humans, little information is available for other PFASs and there are inconsistencies in the literature (EFSA CONTAM Panel 2020). Bioavailability appears to depend on the type of PFAS, the absorption rate of PFASs across the human intestinal barrier and the sex, but variations in the study setup hamper comparability of studies. Furthermore, translation from rodents to humans is known to be challenging due to differences in toxicokinetic parameters between humans and rodents. To that end, alternative human cell-based in vitro methods to study absorption of various PFASs under comparable conditions could greatly add to the evaluation of the toxicokinetics of PFASs in humans.

Well-established intestinal barrier models resembling the intestinal physiology are lacking. Currently, the most widely applied and accepted in vitro model for transport studies is an intestinal barrier model based on the human intestinal epithelial cell (IEC) line Caco-2. The model consists of Caco-2 cells that are cultured in a monolayer on Transwell inserts and differentiated into IECs that display characteristics of in vivo human intestinal epithelial cells. However, the Caco-2 cell-based intestinal barrier model has several limitations when comparing it with the in vivo human intestinal epithelium, like high transepithelial electrical resistance values, presence of only enterocytes and the lack of a mucus layer (Sun et al. 2008). Human induced pluripotent stem cells (hiPSCs)-derived IEC models are promising novel in vitro models, which better reflect human intestinal tissue make-up and functionality than Caco-2 cells. For example, whereas Caco-2 cells have no or limited expression of the most important intestinal CYP enzyme CYP3A4 (Prueksaritanont et al. 1996; Ozawa et al. 2015; Küblbeck et al. 2016), hiPSCs-derived intestinal organoids demonstrate *CYP3A4* mRNA expression and possess CYP3A4 activity (Onozato et al. 2018; Janssen et al. 2021). Moreover, hiPSCs-derived intestinal organoids contain multiple cell types including enterocytes, stem cells, goblet cells, Paneth cells, enteroendocrine cells and mesenchymal cells (Spence et al. 2011; Tamminen et al. 2015; Takayama et al. 2019; Janssen et al. 2021; Grouls et al. 2022). However, the spheric configuration of intestinal organoids poses a practical limitation, as their microarchitecture limits easy access to the apical epithelium to facilitate transport studies. Alternatively, hiPSCs can also be differentiated into IEC layers (Kabeya et al. 2018, 2020; Grouls et al. 2022). This layer model has been shown to also contain multiple cell types, including enterocytes and goblet cells, possesses CYP activity, as well as transporter activity (Kabeya et al. 2018, 2020).

In the current study, we studied the transport of PFASs in hiPSC-derived IEC layers as a model for the human intestinal barrier and compared the data with transport data obtained with the Caco-2 cell model and a commercially available human primary IEC-based model (EpiIntestinal). The EpiIntestinal model is a small intestinal microtissue based on human primary cells, which has been described to appropriately predict human intestinal absorption (Ayehunie et al. 2018; Cui et al. 2020). The hiPSC-derived IEC layers were obtained based on a differentiation method described by Kabeya et al. (2020). Before studying PFAS transport, we extensively characterized the hiPSC-derived IEC layers by evaluating their differentiation during various differentiation stages, followed by evaluation of their cellular make-up, gene expression profiles and transport functionality. Characterization data of hiPSC-derived IEC layers was compared with data obtained from Caco-2 cells and the EpiIntestinal model where applicable. Four PFASs, namely perfluorooctanoic acid (PFOA), perfluorooctane sulfonate (PFOS), perfluorononanoic acid (PFNA) and perfluorohexanesulfonate (PFHxS), were selected for bidirectional transport studies. These substances have been described to currently contribute most to human exposure and in 2020 EFSA established a health based guidance value of 4.4 ng/kg bw for the sum of these four PFASs (EFSA CONTAM Panel 2020). In addition, also hexafluoropropylene oxide-dimer acid (HFPO-DA) was included in this study because it is increasingly used as a replacement for PFOA and other PFASs and is also associated with several health concerns (EPA 2021).

## Materials and methods

### Chemicals

For characterization of transport functionality of the intestinal cell layers, the following model chemicals were used: warfarin, propranolol, quinidine, atenolol, enalapril and acebutolol. The tested PFASs were: perfluorooctanoic acid (PFOA), perfluorooctane sulfonate (PFOS), perfluorononanoic acid (PFNA), perfluorohexane sulfonate (PFHxS) and hexafluoropropylene oxide dimer acid (HFPO-DA). All stocks were prepared in 100% dimethyl sulfoxide (DMSO HybriMax, Sigma, St. Louis, MO). More information about suppliers, purity, catalog numbers and CAS numbers is presented in Supplementary Table 1.

### Caco-2 cell culture

Human colorectal adenocarcinoma cells (Caco-2, HTB-37) were obtained from American Type Culture Collection (ATCC, Manassas, VA). Cells were cultured in a humidified incubator (37°C, 5% CO_2_) in Dulbecco’s modified Eagle’s medium (DMEM) supplemented with 10% heat-inactivated fetal bovine serum (Gibco, Thermo Fisher Scientific, Waltham, MA), 1% non-essential amino acids (Gibco) and 1% penicillin/streptomycin (Sigma). For experiments, Caco-2 cells below passage number 50 were used within 10 passages. They were harvested at 80% confluence using Trypsin/EDTA (0.25%/0.05%; PAN Biotech, Aidenbach, Germany) and seeded at a density of 4 × 10^5^ cells/cm^2^ into inserts with a polycarbonate membrane (0.4 µm pore, Corning, New York, NY). After 21 days of differentiation (using 500 µl cell culture medium in the apical and 1500 µl in the basolateral compartment), monolayer integrity was determined by measuring transepithelial electrical resistance (TEER) with a Millicell ERS Volt-Ohm meter (Millipore, Bedford, MA). Only monolayers exhibiting TEER values exceeding 400 Ω · cm^2^ were used for experiments.

### EpiIntestinal culture

The human EpiIntestinal small intestinal microtissues, consisting of epithelial cells and an underlying lamina propria containing intestinal fibroblasts, were obtained from MatTek Life Sciences (Ashland, USA). Cell culture inserts (8.8 mm ID) were placed in 12-well plates and cells were maintained in 100 µl maintenance medium (MatTek Life Sciences) in the apical compartment and 5 ml maintenance medium in the basolateral compartment upon arrival. The transport studies were performed 1 day after arrival.

### hiPSC culture

The hiPSC cell line (CS83iCTR-33n1) was provided by the Cedars-Sinai Medical Center’s David and Janet Polak Foundation Stem Cell Core Laboratory. These cells had been generated through episomal reprogramming of fibroblasts of a 31-year-old healthy female. The cell line was fully characterized and no karyotype abnormalities were found. hiPSCs were cultured on human embryonic stem cell qualified Matrigel-coated (Corning #354277) cell culture plates in mTeSR1 medium (Stem Cell Technologies, Vancouver, Canada) and passaged using gentle cell dissociation reagent (Stem Cell Technologies).

### hiPSC differentiation into IEC layers and intestinal organoids

For differentiation, hiPSC were dissociated into single cells using Accutase (Stem Cell Technologies) and cultured on human embryonic stem cell qualified Matrigel-coated 24-well plates in mTeSR1 supplemented with 10 µM Y-27632 (Stem Cell Technologies) for 1 day. Human iPSCs were subsequently differentiated into definitive endoderm (DE) by incubation in RPMI1640 medium containing 1% non-essential amino acids (Gibco) and 100 ng/ml Activin A (Cell Guidance Systems, Cambridge, UK), with increasing concentrations of fetal bovine serum (0%, 0.2% and 2% on day 1, 2 and 3, respectively). 50 ng/ml BMP4 (R&D Systems, Minneapolis, MN) was also added during the first day of definitive endoderm formation.

For differentiation into IECs, definitive endoderm cells were subsequently differentiated into intestinal stem cells by changing medium into DMEM/F12 containing 2% fetal bovine serum, 1% GlutaMAX (Gibco) and 250 ng/ml FGF2 (R&D Systems). After 4 days, cells were dissociated using Accutase and seeded onto polyester Transwell inserts (0.4 µm, Corning) coated with Growth Factor Reduced Matrigel #356231. Cells were cultured for 7 days in intestinal differentiation medium (DMEM/F-12 containing 2% fetal bovine serum, 1% non-essential amino acids (Gibco), 1 × B27 (Gibco), 1 × N2 (Gibco), 1% penicillin/streptomycin (Gibco) and 2 mM L-glutamine (Gibco)) supplemented with 20 ng/ml EGF (R&D Systems) and 30 µM Forskolin (Sigma). ROCK inhibitor, y-27632 (final concentration 10 µM; Stem Cell Technologies), was added during the initial 24 h after seeding to reduce cell death. The cells were subsequently cultured for 12 days in intestinal differentiation medium supplemented with 20 ng/ml EGF, 30 µM Forskolin, 5 µM 5-aza-2′-deoxycytidine (Sigma), 20 µM PD98059 (Stem Cell Technologies) and 0.5 µM A-83–01 (Sigma). The medium was refreshed every 2–3 days. Barrier integrity was determined by measuring apical to basolateral translocation of Lucifer yellow (see Supplementary Data for a detailed description).

Definitive endoderm cells were also differentiated into human intestinal organoids (HIOs), as previously described (Janssen et al. 2021). Briefly, definitive endoderm cells were differentiated into hindgut endoderm using FGF4 (R&D Systems) and Chiron99021 (Stemgent). After 4 days, free-floating spheroids were collected and embedded into domes of Matrigel (Corning #354234) and further differentiated using 50 ng/ml EGF (R&D Systems), 100 ng/ml Noggin (R&D Systems) and 500 ng/ml R-spondin-1 (R&D Systems) for 38 days. The medium was refreshed every 2–3 days and HIOs were passaged every 10–14 days. To promote intestinal differentiation, 0.5 µM A-83-01 (Sigma), 20 µM PD98059 (Stem Cell Technologies), 5 µM 5-aza-2’-deoxycytidine (Sigma) and 5 µM DAPT (Sigma) were added to the medium the last 7 days.

### RNA isolation and qPCR

Total RNA was extracted from the hiPSCs-derived IEC layers using the RNeasy Mini Kit (Qiagen, Venlo, The Netherlands). Subsequently, 500 ng RNA was used to synthesize cDNA using the iScript cDNA synthesis kit (Bio-Rad Laboratories, Veenendaal, The Netherlands). Changes in gene expression were determined by real-time PCR on a CFX384 real-time PCR detection system (Bio-Rad Laboratories) using SensiMix (Bioline; GC Biotech, Alphen aan den Rijn, The Netherlands). A standard curve of pooled cDNA samples was used to calculate amplification efficiency, which was for each primer pair within the acceptable range between 90 and 110% (Rogers-Broadway and Karteris 2015). The expression of the gene of interest, calculated using this standard curve, was subsequently normalized to the expression of the housekeeping gene *RPL27*. PCR conditions are described in the Supplementary Data and the used primers are listed in Supplementary Table 2.

### Immunofluorescence staining

hiPSC-derived IEC layers were fixed by gently replacing the medium in the apical compartment with 200 µl room temperature paraformaldehyde (1% v/v in PBS; Thermo Fisher Scientific). For immunocytochemistry, cells were fixed with Carnoy’s fixative (60% ethanol, 30% chloroform, 10% glacial acetic acid and 1 g ferric chloride; all from Sigma) and processed as previously described (van der Hee et al. 2018, 2020). The Transwell inserts were subsequently transferred to wells containing 1 ml of their corresponding fixative and incubated overnight at 4 °C. After fixation, the Transwells were gently cut using a scalpel and processed in 1.5 ml tubes (Eppendorf). For whole mount staining, the Transwells were washed three times in TBS-triton buffer (8.8 g/l NaCl, 6.06 g/l Tris, 0.02% triton X-100, pH 7.4; all from Sigma) and blocked using normal goat serum (5% v/v in TBS-t, #10000C, Thermo-Fisher) for 30 min at RT. The Transwells were then incubated with primary antibodies overnight at 4 °C. After fixation and after each incubation with antibodies or other staining solution, Transwell membranes were washed three times with TBS-t (5 min incubation per wash). After incubation with the first antibody, Transwells were incubated with a secondary antibody (1:400 in TBS-t) for 1 h at RT. Nuclei were stained with 4′6-diamidino-2-phenylindole (DAPI 1 µg/ml in PBS; Sigma-Aldrich) for 10 min at RT. The primary and secondary antibodies are listed in Supplementary Table 3. Transwell membranes were mounted in Diamond anti-fade mountant (Thermo Fisher) on a poly-l-lysine-coated glass slide and a coverslip. All slides were imaged on a DM6 microscope fitted with DFC365 FX camera (Leica), or on a Stellaris 8 confocal point scanning system at 40 × magnification (Leica) and images were processed using Leica Application Suite X software (LAS X, version 3.7.4.23463, Leica).

### RNA isolation for RNA seq and data analysis

To compare whole genome expression of the various intestinal models, RNA was extracted from Caco-2 cells, hiPSC-derived IEC layers or EpiIntestinal microtissues using the RNeasy Mini Kit (Qiagen, Venlo, The Netherlands). Total RNA was extracted from hiPSC-derived organoids using the NucleoSpin RNA isolation kit (Macherey-Nagel, Düren, Germany).

Total RNA was quantified using a Qubit fluorometer (Invitrogen, Life Technologies) and RNA integrity was analyzed using a total RNA Pico chip in an Agilent 2100 Bioanalyzer (Agilent Technologies). Subsequently, RNA Library preparations and RNA sequencing were performed at the Genomics Facility of Wageningen University and Research, Business Unit Bioscience. A detailed description of the RNA Library preparations and RNA seq, and the processing of RNA seq reads is given in the Supplementary Data. RNAseq data were deposited in the NCBI Gene Expression Omnibus under accession number GSE268603.

Multidimensional scaling analysis was performed to visualize multidimensional variation of the differentially expressed genes between the various models and samples. Limma (version 3.50.3) was used to create a multidimensional scaling (MDS) plot. Differential gene expression in the various models was evaluated by comparison with the EpiIntestinal model, a *p* value < 0.01 was considered statistically significantly different based on empirical Bayes moderate t-statistic. Venn diagrams, showing differentially expressed genes in the various models were made using the open access online tool Venny (version 2.1.0; https://bioinfogp.cnb.csic.es/tools/venny/). Additionally, a heatmap was made using Morpheus (https://software.broadinstitute.org/morpheus/) to visualize the differentially expressed genes [showing the respective genes’ counts per million (cpm)] that were specific for one or two models only. Therefore, differentially expressed genes that were shared by all three models (the center of the Venn diagram) were excluded. Gene expression, relative to the EpiIntestinal microtissues, was hierarchically clustered based on a Euclidean distance algorithm.

Gene set enrichment analysis (GSEA; version 4.3.2; Broad Institute, Cambridge, MA) was used to identify enriched or suppressed gene sets (Subramanian et al. 2005). Gene sets were derived from Biocarta, KEGG and WikiPathways pathway databases and ranked based on the normalized enrichments score (NES) (cutoff nominal *p* value < 0.05 and FDR *q* value < 0.25). Only gene sets comprising more than 15 and fewer than 500 genes were taken into account. Statistical significance of GSEA results was determined using 1000 permutations.

### Transport studies

Transport studies were performed in Caco-2 cells, hiPSC-derived IECs and EpiIntestinal microtissues. To that end, Caco-2 cell layers were differentiated for 21 days and hiPSCs were differentiated into IEC layers for 26 days, as described above. EpiIntestinal microtissues were subjected to transport studies 1 day after arrival. Transport studies with the model compounds (warfarin, propranolol, quinidine, atenolol, enalapril and acebutolol) were performed in apical to basolateral, and basolateral to apical direction in HBSS containing 10 µM of each model compound, except for the EpiIntestinal microtissues where only transport of Warfarin and Atenolol was assessed in apical to basolateral direction. For all models, PFAS transport was assessed in apical to basolateral, and basolateral to apical direction using HBSS containing a mixture of PFASs containing 1 µM of each PFAS. See Supplementary Data for a detailed description of the method used for the transport studies of the model compounds and PFASs.

### Quantification of model compounds using LC–MS/MS

For the transport studies of the model compounds, samples of the donor and receiver compartment and cell lysates were analyzed on a Waters Acquity I UPLC (Milford, USA) system. The system was equipped with a Waters Acquity UPLC BEH C18 (100 × 2.1 mm, 1.7 μm) column. The column oven was kept at 60 °C and the temperature of the autosampler was kept at 10 °C. The mobile phase consisted of water (mobile phase A) and 90% methanol (mobile phase B) both containing 1 mM ammonium formate and 0.1% formic acid. The gradient started at 0% B, was kept at 0% B for 1 min, was then linearly increased to 50% B in 2 min and was kept at 50% B for 1 min. Then, the gradient linearly increased to 100% B in 2 min and was kept at 100% B for 2 min. The gradient returned to 0% B within 0.5 min and was kept at this condition for 2.5 min before the next injection. The injection volume used was 5 μl.

Mass spectrometric detection was performed using a Micromass Quattro Ultima mass spectrometer (Waters, Milford, USA) equipped with an electrospray ionization interface (ESI). A capillary voltage of 2.50 kV, a source temperature of 120 °C, a desolvation temperature of 350 °C, a cone gas flow of 194 L/h and a desolvation gas flow of 568 l/h was used. The collision-induced dissociation gas used was argon. Cone voltage and collision energy were optimized by direct infusion for each compound. Parameters for multiple reaction monitoring (MRM) such as ion mode, mass charge (m/z) transitions, cone voltage and collision energy for each compound are described in Supplementary Table 4.

### Quantification of PFASs from transport experiment using LC–MS/MS

Samples of culture medium from the transport experiments were prepared for PFAS measurement by adding 50 μl of the culture medium to 850 μl methanol (Actuall Chemicals, Oss, The Netherlands) containing internal standards [^13^C_4_-PFOA, ^13^C_5_-PFNA, ^18^O_2_-PFHxS, ^13^C_4_-PFOS and ^13^C_3_-HFPO-DA (Wellington Laboratories, Canada)]. After vortexing, the samples were centrifugated at maximum speed (20,817×*g*) for 10 min at 4 °C. PFAS concentrations were determined in the supernatant using LC–MS/MS analysis. LC separation was performed using either a Shimadzu UHPLC system containing: two pumps (LC 20AD xr); a column oven (Shimadzu CTO-20AC); a pump switch (Shimadzu FCV-11AL); a degasser (Shimadzu DGU-20A3); and a sample tray holder (Shimadzu SIL-20 AC XR model) (Shimadzu Corporation, Kyoto, Japan) or a Sciex UHPLC system containing: two pumps (ExionLC AD); column oven (ExionLC AC); controller (ExionLC); degasser (ExionLC); and sample tray holder (Exion AD) (Sciex Framingham, MA, USA). Optimized PFAS measurement protocols for each system were used. To separate the PFASs, an Acquity BEH-C18 analytical column (50 × 2.1 mm i.d., 1.7 μm, Waters, Milford, MA, USA) (Shimadzu system) or Luna Omega PS C18 analytical column (100 Å, 100 × 2.1 mm i.d., 1.6 µm, Phenomenex, Torrance, CA, USA) (Sciex system) was used at a column temperature of 35 °C (Shimadzu system) or 40 °C (Sciex system). Additionally, a symmetry C18 analytical column (50 × 2.1 mm i.d., 5 µm, Waters, Milford, MA, USA) (Shimadzu system) or Gemini C18 analytical column (110 Å, 50 × 3 mm i.d., 3 µm, Phenomenex, Torrance, CA, USA) (Sciex system) was used as an isolator column, which was placed between the pump and the injector valve to isolate and delay interferences out of the LC system. The mobile phase consisted of 2 mM (Shimadzu system) or 20 mM (Sciex system) ammonium acetate (Merck Millipore, Darmstadt, Germany) in Milli-Q water (prepared using a Milli-Q system with a resistivity of at least 18.2 M Ω cm^−1^ (Merck Millipore)) (Shimadzu system) or Ultra LC/MS grade water (Actu-All Chemicals, Oss, The Netherlands) (Sciex system) (mobile phase A) and Acetonitrile ULC/MS grade (Actu-All Chemicals, Oss, The Netherlands) (mobile phase B). The injection volume used was 20 μl. The chromatographic gradient was operated at a flow rate of 0.3 ml/min (Shimadzu system) or 0.8 ml/min (Sciex system) starting from 25% (Shimadzu system) or 15% (Sciex system) mobile phase B in the first 0.1 (Shimadzu system) or 1.0 (Sciex system) minute followed by a linear increase to 100% (Shimadzu system) or 98% (Sciex system) B in 6 min which was held for 2.5 (Shimadzu system) 0.5 (Sciex system) minutes. Then, the gradient returned to 25% (Shimadzu system) or 15% (Sciex system) B within 0.1 min and was held for 3.9 (Shimadzu system) or 0.7 (Sciex system) minutes to equilibrate before the next injection, resulting in a total run time of 12.5 (Shimadzu system) or 8.3 (Sciex system) minutes. Mass spectrometric detection was carried out by MS/MS using a Sciex QTRAP 5500 (Shimadzu system) or Sciex QTRAP 7500 (Sciex system) system (Sciex, Framingham, MA, USA) in negative electrospray ionization (ESI-) mode. The following conditions were used: an ion spray voltage (IS) of − 4500 V (Shimadzu system) or − 1500 V (Sciex system); curtain gas (CUR) of 30 l/h (Shimadzu system) or 45 psi (Sciex system); a source temperature (TEM) of 350 °C (Shimadzu system) or 400 °C (Sciex system); gas 1 (GS1) of 55 l/h (Shimadzu system) or 40 psi (Sciex system); gas 2 (GS2) of 60 l/h (Shimadzu system) or 80 psi (Sciex system); and collision gas (CAD) high (Shimadzu system) or 9 (Sciex system). The PFASs were fragmented using collision induced dissociation (CID) using argon as target gas. The analyses were performed in multiple reaction monitoring (MRM) mode, using two mass transitions per component selected based on the abundance of the signal and the selectivity of the transition. In Supplementary Table 5 and 6 information on the MRM transitions, declustering potential (DP), entrance potential (EP), collision energy (CE) and cell exit potential (CXP) are presented for the Shimadzu and Sciex system. Data were acquired using Analyst software (Shimadzu system) or SciexOS software (Sciex system) and processed using MultiQuantTM software (Sciex, Framingham, MA, USA).

### Statistical analysis

Data are presented as mean ± SEM. A one-way ANOVA, followed by Bonferroni’s post hoc multiple comparison test was used for comparisons of the *P*_app_ values of model compound and PFAS transport between the three different models. An unpaired Studen’s *t* test was used for comparison of the P_app_ values of basolateral to apical model compound transport between hiPSC-derived IECs and Caco-2 cells. *p* < 0.05 was considered statistically significant. Prism software (version 9.2.0; GraphPad, San Diego, CA) was used for statistical analysis.

## Results

### Differentiation and characterization of hiPSC-derived intestinal epithelial cell layers

**Fig. 1:**
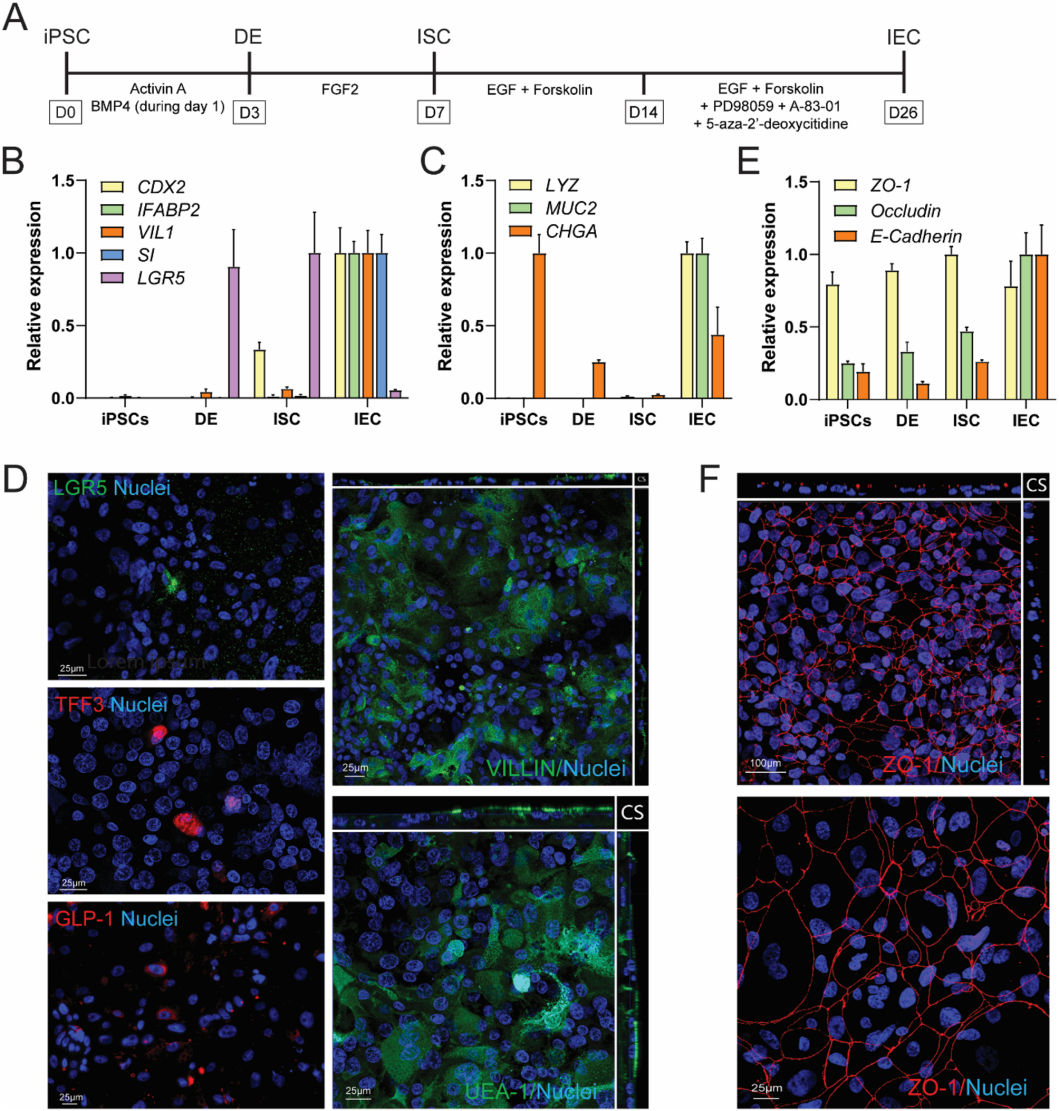
Differentiation of hiPSCs into intestinal epithelial cell (IEC) layers. **A** Schematic procedure for the differentiation of hiPSCs into the IEC layers. **B** Relative expression of intestinal differentiation and stem cell markers during the course of the differentiation from hiPSCs up to IEC layers. The highest expression level of each gene was set at one. Data are presented as mean values ± SEM from two independent experiments with triplicate wells. **C** Relative expression of markers of Paneth cells (*LYZ*), goblet cells (*MUC2*) and enteroendocrine cells (*CHGA*) during the course of the differentiation from hiPSCs up to IEC layers. The highest expression level of each gene was set at one. Data are presented as mean values ± SEM from two independent experiments with triplicate wells. **D** Representative immunofluorescent pictures of IEC layers stained for various intestinal differentiation markers. Nuclei were counterstained with 4′6-diamidino-2-phenylindole (DAPI). LGR5: leucine-rich repeat-containing G-protein-coupled receptor 5, TFF3: trefoil factor 3, GLP-1: glucagon-like peptide 1, UEA-1: *Ulex europaeus* agglutinin-1. The VIILIN and UEA-1 images show a cross section (CS) and a maximum intensity projection of all imaged layers. **E** Relative mRNA expression of the tight junctional proteins zonula occludens 1 (ZO-1) and occludin, and the adherens junction protein E-cadherin during the course of the differentiation from hiPSCs up to the IEC layers. The highest expression level of each gene was set at one. Data are presented as mean values ± SEM from two independent experiments with triplicate wells. **F** Representative immunofluorescent pictures of IEC layers stained for ZO-1. Both images show maximum intensity projection of all imaged layers. Nuclei were counterstained with DAPI

The differentiation procedure of the human hiPSC line CS83iCTR-33n1 into definitive endoderm was based on a previous report (Janssen et al. 2021). Definitive endoderm was subsequently differentiated into IEC layers based on methods described by Kabeya et al. (2018, 2020) (Fig. 1A). To assess the differentiation procedure, terminally differentiated hiPSC-derived IEC layers were characterized using gene expression measurements and immunohistochemical evaluation. The expression levels of the differentiation markers were normalized to the expression of the housekeeping gene *RPL27*, which remained stable throughout all phases of differentiation phases in both independent experiments (Supplementary Fig. 1A). The intestinal differentiation markers *CDX2* (intestinal transcription factor), *IFABP2* (cellular protein specific for mature enterocytes), *VIL1* (major component of brush border cytoskeleton specific for enterocytes) and *SI* (a glucosidase enzyme located on the brush border of the small intestine) were well expressed in the IEC phase (Fig. 1B; Supplementary Table 7). Villin1 expression could also readily be detected as confirmed by immunofluorescence (Fig. 1D). The intestinal stem cell marker *LGR5* gradually increased over time with the highest expression in the ISC phase (average Ct value of 27.1; Fig. 1B). Despite the highest expression of LGR5 in the ISC phase, some expression of LGR5 was still present in the IEC phase and could also still be visualized in this phase (Fig. 1D; Supplementary Table 7). In addition to enterocytes and stem cells, the IEC layer also contained Paneth cells, goblet cells and enteroendocrine cells as evidenced by an upregulation of *LYZ*, *MUC2* and *CHGA* in the IEC phase, respectively (Fig. 1C; Supplementary Table 7). These findings were corroborated by immunofluorescence analysis using GLP1 to visualize enteroendocrine cells, the lectin Ulex Europaeus Agglutinin I (UEA-1), which is known to stain Paneth cells and goblet cells including secreted mucins, and Trefoil Factor 3 (TFF3), staining glycosylated mucus proteins present in mature goblet cells (Fig. 1D). The IECs grow in a confluent layer, which was confirmed to be a monolayer by evaluation of a cross section of the IEC layer (Fig. 1D). The expression of the tight junction protein occludin and the adherens junctions protein E-cadherin gradually increased over time during differentiation with the highest expression in the IEC phase (Fig. 1E; Supplementary Table 7). The tight junction protein ZO-1 was highly expressed throughout all phases of differentiation (Fig. 1E, Supplementary Table 7). The presence of ZO-1 was also visualized using immunofluorescence staining (Fig. 1F). In addition, ZO-1 staining (Fig. 1F), VILLIN staining and UEA-1 staining (Fig. 1D) were mainly located at the apical surface, indicating polarization of the IEC layer with formation of tight junctions, a brush border and a mucus layer on the luminal side. Gene expression data from each independent experiment are presented in Supplementary Fig. 1B. While most differentiation markers were well expressed in the IEC phase (Supplementary Table 7), there was some variability in the expression of *LGR5*, *CHGA*, *ZO-1* throughout the differentiation process. The TEER remained constant from day 19 of the differentiation protocol onwards, amounting to ~ 360 Ω cm^2^, and there was no quantifiable apical to basolateral transport of lucifer yellow in terminally differentiated IECs, indicating a proper barrier integrity (data not shown).

### Transcriptomics of four intestinal models

**Fig. 2:**
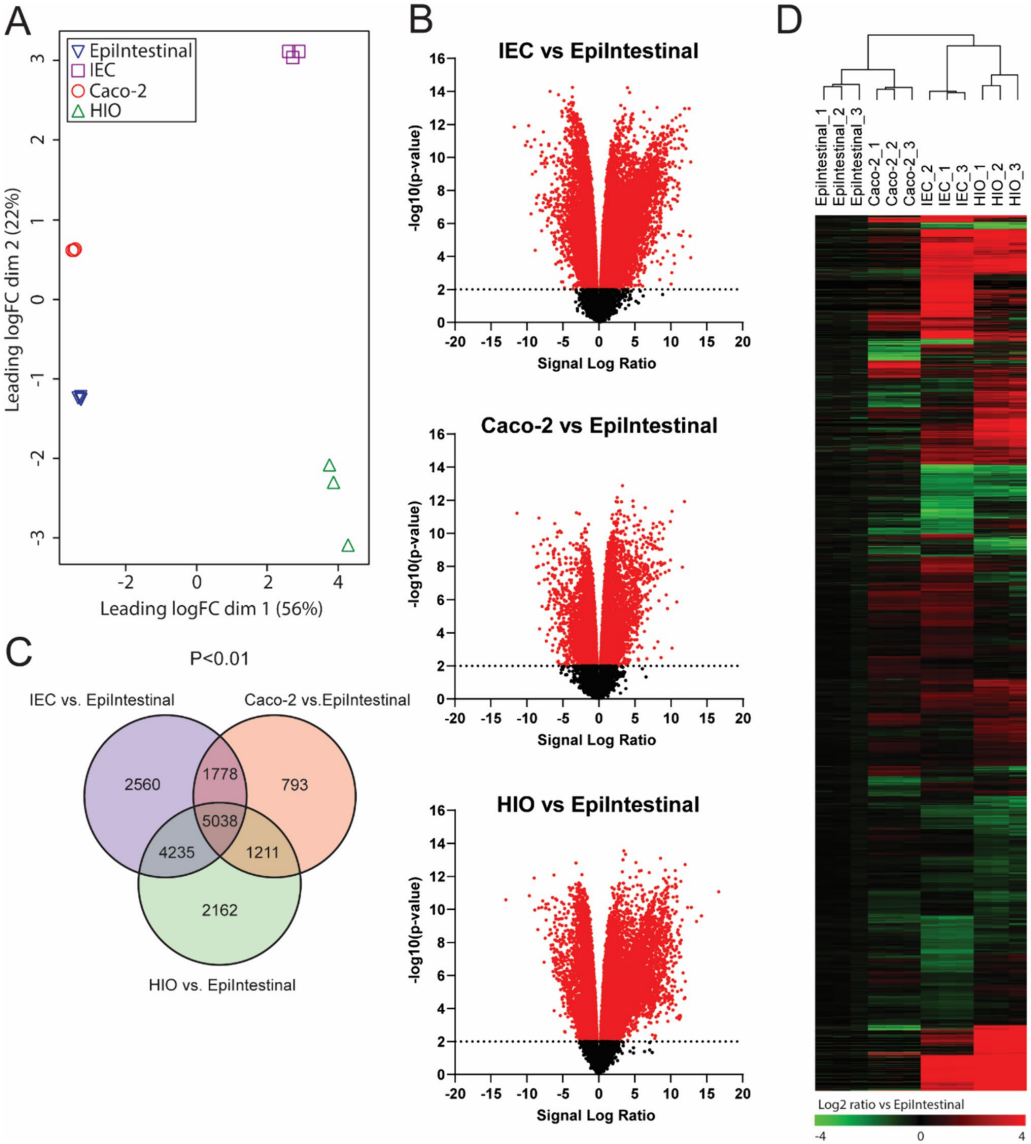
Transcriptomics analysis of the EpiIntestinal model, hiPSC-derived IEC layers, Caco-2 cell layers and hiPSC-derived HIOs.** A** Multidimensional scaling plot of transcriptome-wide gene expression in the four intestinal models. Numbers in parentheses represent the percentage of total variance explained by the first and second dimension. **B** Volcano plots showing relative changes in gene expression (expressed as signal log_2_ ratio, *x*-axis) plotted against statistical significance (expressed as − log10 *p* value of empirical Bayes moderated *t* statistic p value, *y*-axis) of the hiPSC-derived IEC layers vs. EpiIntestinal model, Caco-2 cell layers vs. EpiIntestinal model and hiPSC-derived HIOs vs. EpiIntestinal model. Dotted line represents cutoff of *p* < 0.01. **C** Venn diagrams showing the number of differentially expressed genes (*p* < 0.01) in the hiPSC-derived IEC layers, Caco-2 cell layers and hiPSC-derived HIOs compared to the EpiIntestinal model. **D** Heatmap obtained upon hierarchical clustering of genes that were differentially expressed in either one or two models, as compared to the EpiIntestinal model. Expression is normalized against average expression of the EpiIntestinal model (arbitrarily set at zero), showing Log2 ratio of expression in the hiPSC-derived IEC layers, Caco-2 cell layers and hiPSC-derived HIOs versus expression in the EpiIntestinal model

**Fig. 3:**
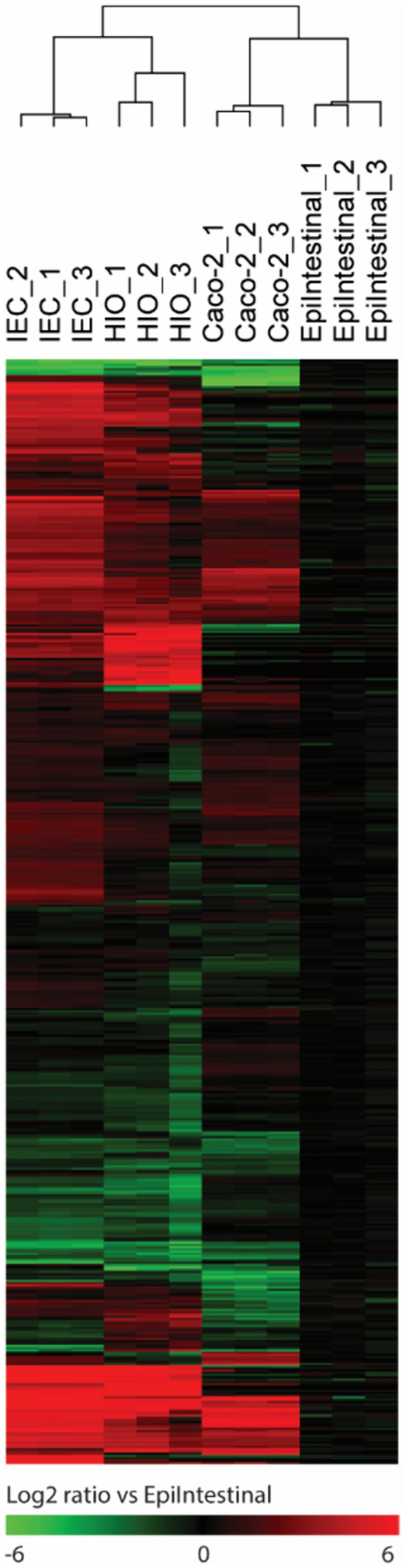
Heatmap obtained upon hierarchical clustering of the gene expression data of 492 intestine-specific genes in the EpiIntestinal model, hiPSC-derived IEC layers, Caco-2 cell layers and hiPSC-derived HIOs. Expression is normalized against average expression of the EpiIntestinal model (arbitrarily set at zero), showing Log2 ratio of expression in the hiPSC-derived IEC layers, Caco-2 cell layers and hiPSC-derived HIOs versus expression in the EpiIntestinal model

Transcriptomics analysis was performed to assess the base level gene expression profiles of the following four intestinal models: hiPSC-derived IEC layers, Caco-2 cell layers, hiPSC-derived HIOs, and a human primary model for the intestine (EpiIntestinal). After normalization and filtering of lowly expressed genes, multidimensional scaling (MDS) analysis was employed to compare global gene expression patterns of the four intestinal models. The MDS plot shows that the replicate samples of each intestinal model cluster together indicating low variability within the sample groups (Fig. 2A). The biggest source of variation was explained by dimension 1 (56%), which showed a clear separation between the hiPSC-derived IECs and HIOs, and the Caco-2 cell layers and EpiIntestinal model. Subsequently, differentially expressed genes were selected using the human primary model for the intestine (EpiIntestinal) for benchmarking. When applying a statistical cutoff of *p* < 0.01 (empirical Bayes moderated t-statistic), 8,820 genes were differentially expressed in the Caco-2 cells, 12,646 genes in the hiPSC-derived HIOs and 13,611 genes in the hiPSC-derived IECs, of the total of 19,619 genes (Fig. 2B). A Venn diagram of these differentially expressed genes shows that there was an overlap of 5,038 genes that were differentially expressed in all three intestinal models, when compared to the EpiIntestinal model. The number of genes that were statistically differentially expressed in only one model compared to the EpiIntestinal model was higher for the hiPSC-derived models (2560 genes in hiPSC-derived IECs and 2162 genes in HIOs) than for the Caco-2 cell layers (793 genes) (Fig. 2C). Next, the 12,739 genes that were differentially expressed in either one or two models, as compared to the EpiIntestinal model, were hierarchically clustered and presented in a heatmap (Fig. 2D). Like in the MDS plot, the heatmap also shows little variation in the gene expression patterns of the technical replicates suggesting robust differentiation methods of Caco-2 cell layers and hiPSC-derived HIOs and IEC layers. Furthermore, the heatmap clearly shows the differential expression between the Caco-2 cell layers, hiPSC-derived IEC layers and the hiPSC-derived HIOs versus the EpiIntestinal model, but it also shows apparent differences between the hiPSC-derived models and the Caco-2 cell layers. In these models, several differentially expressed genes showed an opposing gene expression when compared to the EpiIntestinal model. Subsequently, a list of 698 intestine-specific genes was obtained from the Human Protein Atlas that have been annotated to be enriched or enhanced in the intestine (Uhlén et al. 2015). 492 (70,5%) of these genes were also expressed in our data set, each having an expressing level greater than approximately ten counts in at least one of the triplicates. These genes were hierarchically clustered and presented in a heatmap again using the EpiIntestinal model as a benchmark (Fig. 3). When comparing the number of differentially expressed intestine-specific genes between the Caco-2 cell layers, hiPSC-derived IEC layers and the hiPSC-derived HIOs versus the EpiIntestinal model, it was found that the hiPSC-derived IEC model has the largest number of differentially expressed intestine-specific genes with a log twofold change > 1 and the smallest number of differentially expressed intestine-specific genes with a log twofold change > − 1 (Table 1). This suggests that of the four intestinal models, the hiPSC-derived IEC layers have the highest expression of intestine-specific genes.

**Table 1:** Number of significantly differentially expressed intestine-specific genes as compared to the EpiIntestinal model

	IEC	Caco-2	HIO
Significantly differentially expressed genes	393	308	365
Log2 FC > 1	255	136	196
Log2 FC < − 1	73	90	114

**Fig. 4:**
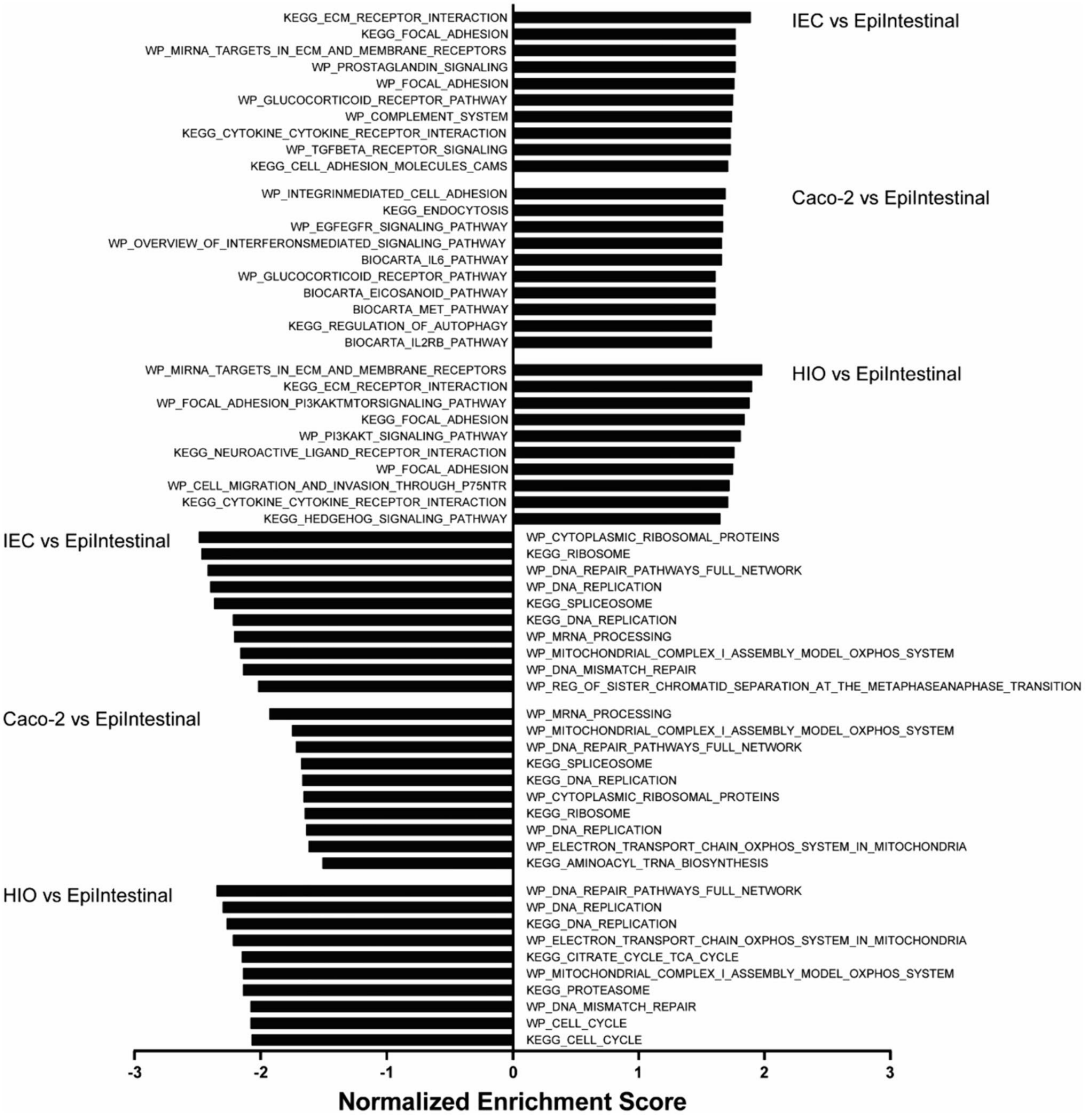
Top ten gene sets induced or repressed in hiPSC-derived IEC layers, Caco-2 cell layers and hiPSC-derived HIOs compared to the EpiIntestinal model. The top ten gene sets were determined using gene set enrichment analysis. Gene sets were ranked according to the normalized enrichment score (NES)

To obtain insight into the biological function of the differentially expressed genes in the three intestinal models in comparison with the EpiIntestinal model, gene set enrichment analysis (GSEA) was performed (Fig. 4). Various pathways related to cytokine receptor interaction were enriched in both hiPSC-derived IEC and HIO models, and the IL6 and IL2RB pathways were enriched in the Caco-2 model, indicating higher expression of genes related to immune function and signaling in all three intestinal models as compared with the EpiIntestinal model. In addition, all three models had elevated expression of gene sets related to cell–matrix adhesion. When comparing pathways with a lower expression, the three models shared lower expression of gene sets related to DNA replication and DNA repair pathways. In addition, in the hiPSC-derived IEC layers and Caco-2 cell layers, there was lower expression of gene sets related to ribosome and RNA processing, whereas in the hiPSC-derived HIOs more pathways related to the cell cycle had a lower expression as compared to the EpiIntestinal model.

**Fig. 5:**
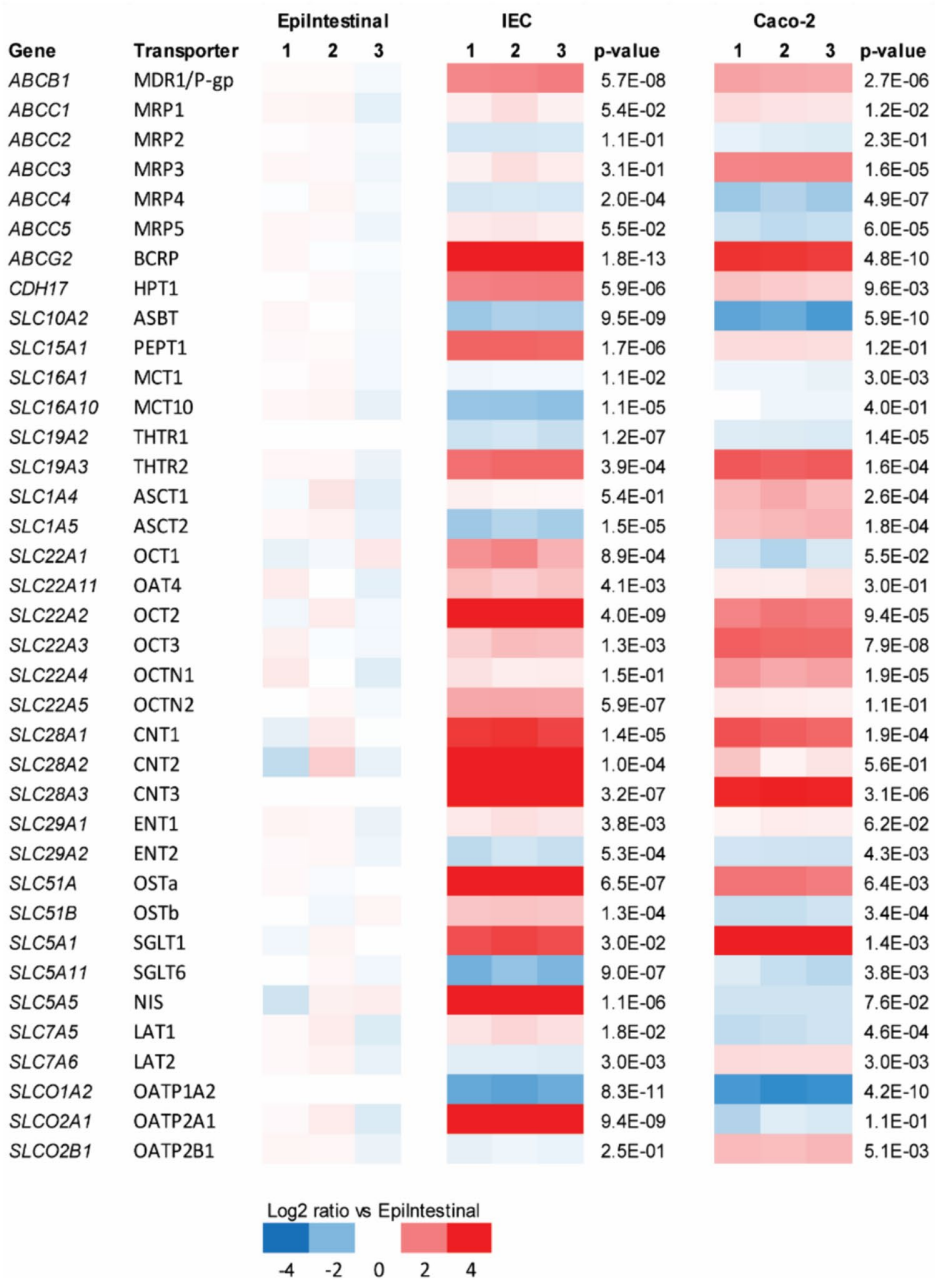
Comparative heatmap analysis on gene expression of intestinal transporters in the EpiIntestinal model, hiPSC-derived IEC layers and Caco-2 cell layers. Expression is normalized against average expression of the EpiIntestinal model (arbitrarily set at zero), showing Log2 ratio of expression in the hiPSC-derived IEC layers and Caco-2 cell layers versus expression in the EpiIntestinal model

In the current study, the main aim was to study the transport of PFASs across the intestinal barrier in the three intestinal epithelial cell layer models. To that end, gene expression analysis specifically focused on comparison of gene expression levels of the most important intestinal transporters in these three models, as visualized in a heatmap (Fig. 5). The heatmap illustrates significant variation between the three different intestinal epithelial cell layer models for the selected genes. For instance, the gene expression of *SLC1A5* and *SLC7A6* was higher in the Caco-2 cell layers, but it was significantly lower in the hiPSC-derived IEC layers as compared to the EpiIntestinal model. In addition, mRNA levels of *SLC51B* and *SLC7A5* were elevated in hiPSC-derived IEC layers, but reduced in the Caco-2 cell layers in comparison with the EpiIntestinal model. Interestingly, out of the 37 selected intestinal transporter genes, 8 genes (*ABCG2*, *SLC19A3*, *SLC22A2*, *SLC22A3*, *SLC28A1*, *SLC28A3*, *SLC51A* and *SLC5A1*) were highler expressed and 6 genes (*ABCC4*, *SLC10A2*, *SLC19A2*, *SLC29A2*, *SLC5A11* and *SLCO1A2*) were found to be lower expressed in the Caco-2 and hiPSC-derived IEC models as compared to the EpiIntestinal model. Taken together, these data suggest that the models could have different active transport properties based on the gene expression levels of the transporters.

### Transport of model compounds

**Fig. 6:**
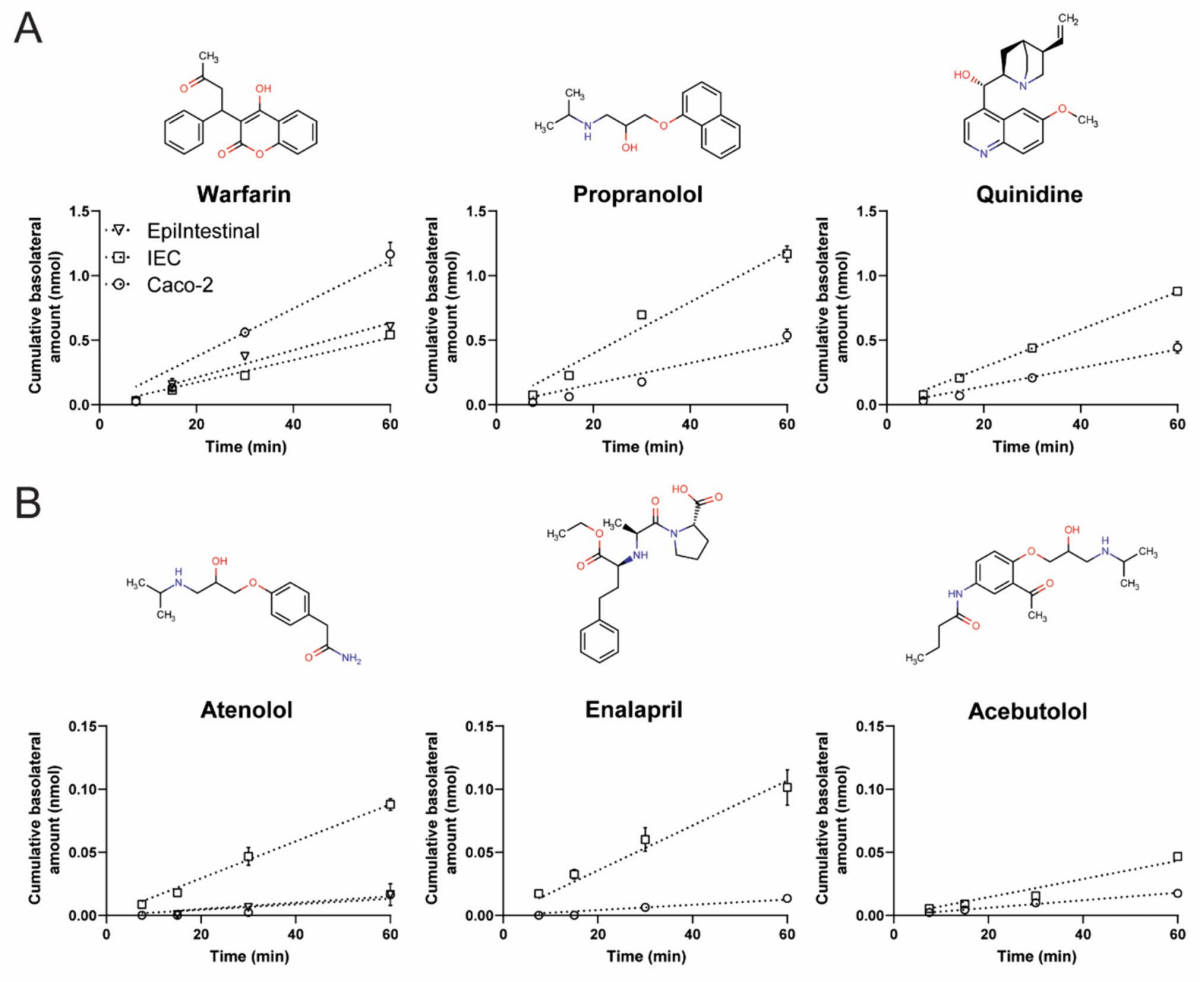
Model compound absorption (A–B direction) in the EpiIntestinal model, hiPSC-derived IEC layers and Caco-2 layers. **A** Cumulative amount of high-permeable compounds in the basolateral compartment during the course of the transport study upon apical exposure to 10 µM of the model compounds at *t* = 0. Samples were taken from the basolateral compartment at settled time points and model compounds were quantified using LC/MS–MS. **B** Cumulative amount of low permeable compounds in the basolateral compartment during the course of the transport study upon apical exposure to 10 µM of the model compounds at *t* = 0. Samples were taken from the basolateral compartment at settled time points and model compounds were quantified using LC/MS–MS. In the EpiIntestinal model, only transport studies for warfarin and atenolol were conducted. Data are mean values ± SEM from triplicate wells

Transport studies were performed in the hiPSC-derived IEC layers, in the primary model for the intestine (EpiIntestinal), and in the Caco-2 cell layers. Transport in hiPSC-derived HIOs was not evaluated due to their microarchitecture that limits easy access to the apical epithelium. To characterize the transport properties of the hiPSC-derived IEC layers, the transport of six model compounds was assessed. Model compounds were selected based on known human absorption data and on the availability of permeability data in the EpiIntestinal model. Based on the biopharmaceutics classification system (BCS), which classifies drugs based on their solubility and permeability, three model compounds with a high permeability (warfarin, propranolol and quinidine) were selected and three model compounds with a low permeability (atenolol, enalapril and acebutolol). The EpiIntestinal model has been described to appropriately predict human intestinal absorption (Ayehunie et al. 2018). Transport studies were conducted in the EpiIntestinal model for warfarin and atenolol in apical to basolateral (A–B) direction (Fig. 6A, B; Table 2), to assess reproducibility of the data for these compounds as described in literature. While warfarin transport was slightly lower in our experiments than reported in literature, the same trends were observed (i.e., high transport for warfarin versus low transport for atenolol), so for comparisons of the apparent permeability coefficients (*P*_app_) between the three models, the data from literature were used (Ayehunie et al. 2018).

**Table 2:** Comparison of model compound absorption in the EpiIntestinal, hiPSC-derived IEC and Caco-2 models

	EpiIntestinal	EpiIntestinal^1^	hiPSC-derived IEC	hiPSC-derived IEC^2^	Caco-2	*F*_a_ (%)^1^	BCS class^1^
	Average *P*_app_ A–B (× 10^–6^ cm/s)	Average *P*_app_ A–B (× 10^–6^ cm/s)	Average *P*_app_ A–B (× 10^–6^ cm/s)	Average *P*_app_ A–B (× 10^–6^ cm/s)	Average *P*_app_ A–B (× 10^–6^ cm/s)		
Warfarin	27.09 ± 0.89	36.7 ± 16.8^a^	15.96 ± 0.87^a^	Nd	17.85 ± 1.39^a^	100	1
Propranolol	Nd	9.4 ± 5.4^b^	26.71 ± 1.40^a^	Nd	11.80 ± 1.07^a,b^	90	1
Quinidine	Nd	7.9 ± 2.7^a^	22.33 ± 0.40^b^	Nd	9.87 ± 1.01^a^	80	2
Atenolol	0.49 ± 0.26	0.5 ± 0.2^a^	2.12 ± 0.11^b^	Nd	0.30 ± 0.05^a^	50	3
Enalapril	Nd	0.4 ± 0.2^a^	2.95 ± 0.41^b^	1.93 ± 0.46	0.28 ± 0.04^a^	40	3
Acebutolol	Nd	0.4 ± 0.1^a^	1.95 ± 0.02^b^	2.35 ± 0.88	0.42 ± 0.03^a^	40	3

Different letters indicate statistically significant differences at *p* < 0.05 per compound

*Nd* not determined, *F*_*a*_ fraction absorbed in humans

^1^Data obtained from Ayehunie et al. (2005)

^2^Data obtained from Kabeya et al. (2020)

Of the high-permeable drugs, *P*_app_ values of A–B transport of warfarin were not significantly different between the three models, but those of A–B transport of propranolol were significantly higher in the hiPSC-derived IEC layer model than in the EpiIntestinal model. Also, the *P*_app_ values of A–B transport of quinidine were higher in the hiPSC-derived IEC layer model than in both the EpiIntestinal model and Caco-2 cell layers (Fig. 6A; Table 2). The *P*_app_ values of all low permeable drugs were significantly higher in the hiPSC-derived IEC layer model as compared to Caco-2 cell layers and the EpiIntestinal model (Fig. 6B; Table 2). There was a general trend of higher *P*_app_ values in the hiPSC-derived IEC model, but the A–B transport of low- and high-permeable compounds can be clearly discriminated. The A–B transport of enalapril and acebutolol in hiPSC-derived IEC layers has been assessed previously (Kabeya et al. 2020) and showed comparable *P*_app_ values as compared to our hiPSC-derived IEC layers, indicating that these higher *P*_app_ values are a characteristic of the model (Table 2). The transport of the model compounds was also assessed in basolateral to apical (B–A) direction (Supplementary Fig. 2), again showing significantly higher P_app_ values for almost all model compounds in the hiPSC-derived IEC layers as compared to Caco-2 cell layers. The exception was the lower P_app_ value for warfarin in hiPSC-derived IEC layers, for which the B–A transport was higher across the Caco-2 cell layers compared to the hiPSC-derived IEC layers (Supplementary Table 8). To further characterize the transport properties of the hiPSC-derived IEC layer model, the efflux ratio (Papp B–A/Papp A–B) of the model compounds was calculated and compared with the efflux ratio derived from the Caco-2 cell model (Table 3). According to the FDA guidance (US Food and Drug Administration 2020), any investigational drug with an efflux ratio greater than 2 is indicative of a potential substrate of active efflux transporters. The mean efflux ratio for all model compounds, except acebutolol, were below 2, suggesting no involvement of active efflux transporters which is in accordance to information from the literature for these drugs. For acebutolol, the efflux ratio was 7.37 in the Caco-2 cell layers and 3.67 in the hiPSC-derived IEC layers, suggesting active efflux transport of acebutolol in both models. Indeed, acebutolol has been described to be a substrate of the efflux transporter P-glycoprotein (Terao et al. 1996). Taken together, the hiPSC-derived IEC layer model can clearly discriminate between low- and high-permeable compounds, and shows the presence of active transport.

**Table 3:** Mean efflux ratio of model compounds in the hiPSC-derived IEC and Caco-2 models

	IEC	Caco-2
Warfarin	1.20	1.37
Propranolol	0.67	0.69
Quinidine	1.14	1.31
Atenolol	0.92	1.09
Enalapril	1.25	1.55
Acebutolol	3.67	7.37

### Transport of PFASs

**Fig. 7:**
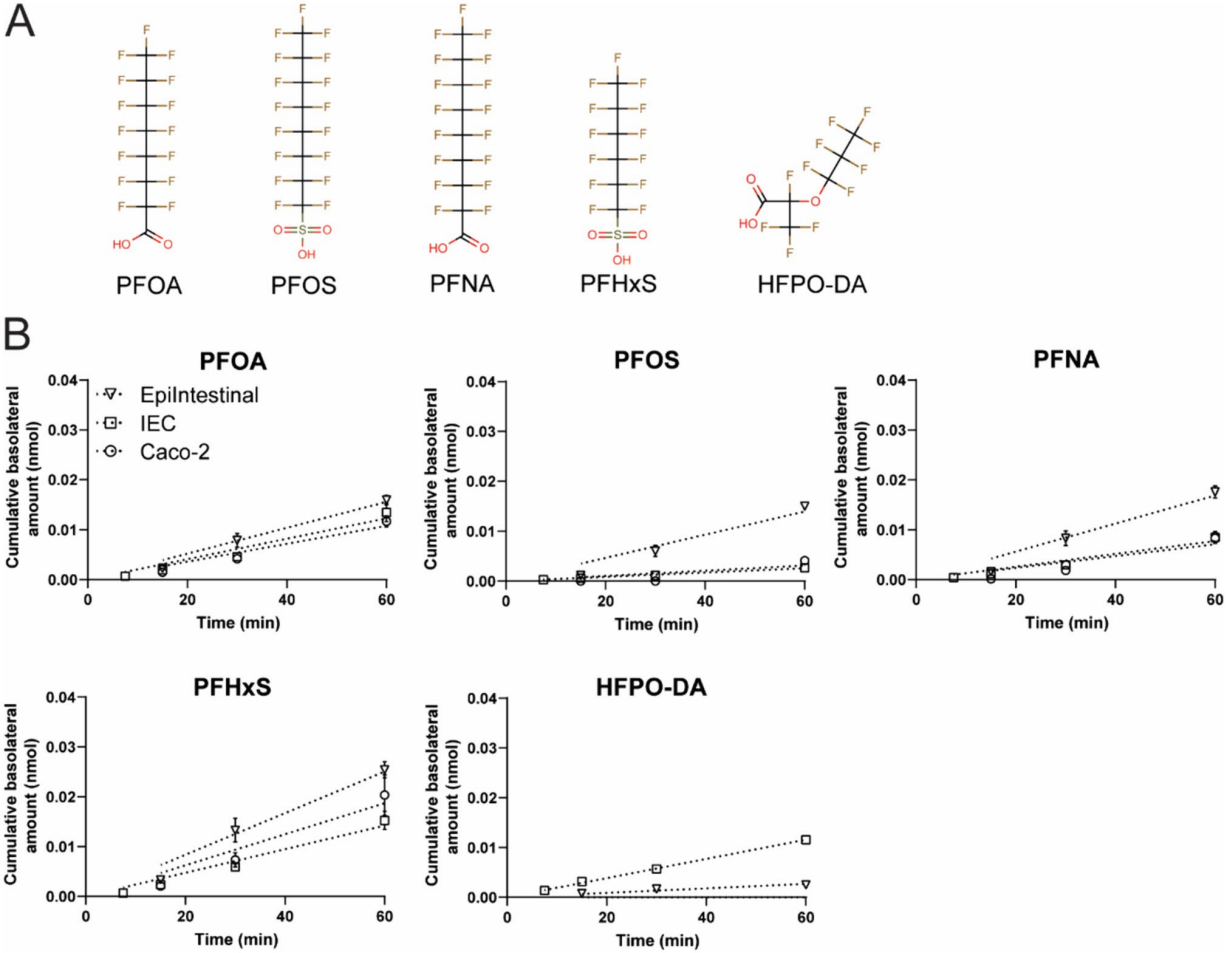
PFASs absorption (A–B direction) in the EpiIntestinal model, hiPSC-derived IEC layers and Caco-2 layers. **A** Chemical structures of the PFASs tested in the present study. *PFOA* perfluorooctanoic acid, *PFOS* perfluorooctane sulfonate, *PFNA* perfluorononanoic acid, *PFHxS* perfluorohexane sulfonate, *HFPO-DA* hexafluoropropylene oxide-dimer acid. **B** Cumulative amount of PFOA, PFOS, PFNA, PFHxS and HFPO-DA in the basolateral compartment during the course of the transport study upon apical exposure to a mixture containing 1 µM of each PFAS. Samples were taken from the basolateral compartment at settled time points and quantified using LC/MS–MS. Data are mean values ± SEM from triplicate wells

The main aim of the current study was to get insight into the transport of some well-known PFASs across the human intestinal barrier in vitro. To that end, the PFASs, PFOA, PFOS, PFNA, PFHxS and HFPO-DA were selected for bidirectional studies (Fig. 7A).

PFAS transport was evaluated in the hiPSC-derived IEC layers, Caco-2 cell layers and in the EpiIntestinal model. Mass balance calculations showed a general recovery of PFASs between 75 and 100% in all three models (Supplementary Fig. 3), indicating some adsorption to the plasticware or degradation of the PFASs. Specifically for PFOS, it was apparent that there was a higher recovery in the cell lysate fractions. The *P*_app_ values of PFAS transport in the hiPSC-derived IEC model in the A–B direction were in the following order: PFHxS > PFOA > HFPO-DA > PFNA > PFOS (Fig. 7B). The order of PFAS transport was highly similar between all models, but differences in the *P*_app_ values were noted. In general, the *P*_app_ values were highest in the EpiIntestinal model (ranging from 1.16 to 12.48 × 10^–6^ cm/s) and lowest in the hiPSC-derived IEC model (ranging from 0.63 to 3.95 × 10^–6^ cm/s) (Fig. 7B**; **Table 4). Transport in B–A direction was also studied (Supplementary Fig. 4; Supplementary Table 9) and generally reflected the results of transport in the A–B direction. The permeability of PFOS was relatively low compared to the transport of the other PFASs with only small differences between the three intestinal models, with the permeability being highest in the EpiIntestinal model in the A–B and B–A directions (Fig. 7B; Supplementary Fig. 4). Interestingly, there was limited to no transport of HFPO-DA in the EpiIntestinal and Caco-2 cell models, whereas transport could be readily detected in the hiPSC-IEC model (Fig. 7B; Supplementary Fig. 4). The efflux ratios were comparable between the three models and were all < 2, suggesting no active transport of PFASs (Table 5). Overall, PFHxS transport was highest in all models and HFPO-DA and PFOS transport was rather limited depending on the intestinal model.

**Table 4:** Apparent permeability (*P*_app_) values of A–B transport of PFASs in the EpiIntestinal, hiPSC-derived IEC and Caco-2 models

	EpiIntestinal	IEC	Caco-2
	Average *P*_app_ A–B (× 10^–6^ cm/s)	Average *P*_app_ A–B (× 10^–6^ cm/s)	Average *P*_app_ A–B (× 10^–6^ cm/s)
PFOA	7.31 ± 0.43^a^	3.27 ± 0.45^b^	4.23 ± 0.41^b^
PFOS	5.97 ± 0.05^a^	0.63 ± 0.08^b^	2.88 ± 0.20^c^
PFNA	8.48 ± 0.57^a^	1.95 ± 0.27^b^	4.55 ± 0.56^c^
PFHxS	12.48 ± 0.78^a^	3.95 ± 0.47^b^	8.78 ± 1.76^a,b^
HFPO-DA	1.16 ± 0.19^a^	3.09 ± 0.09^b^	ND^c^

ND: not determined; could not be calculated since HFPO-DA could not be detected in the basolateral compartment

Different letters indicate statistically significant differences at p < 0.05 per compound

**Table 5:** Efflux ratio of PFASs in the EpiIntestinal, hiPSC-derived IEC and Caco-2 cell models

	EpiIntestinal	IEC	Caco-2
PFOA	0.96	1.62	1.36
PFOS	0.55	1.31	0.37
PFNA	0.89	1.48	0.69
PFHxS	1.13	1.70	1.33
HFPO-DA	0.90	1.45	ND

ND: not determined; could not be calculated since HFPO-DA could not be detected

## Discussion

In the present study, we aimed to get insight into the transport of PFASs across the intestinal barrier using hiPSC-derived IEC layers in comparison with the human primary IEC-based EpiIntestinal model, which has been described to closely resemble human small intestinal tissue (Ayehunie et al. 2018), and the human colonic adenocarcinoma cell line Caco-2, which is commonly used for permeability studies. Characterization of the hiPSC-derived IEC layers showed that they had a polarized epithelium, consisting of enterocytes, intestinal stem cells, enteroendocrine cells, goblet cells and Paneth cells and thus mimic the cellular make-up of intestinal tissue in vivo. RNA sequencing was performed to benchmark the hiPSCs-derived IEC and Caco-2 layer models against the human EpiIntestinal model. All models showed a significant amount of differentially regulated genes compared to the EpiIntestinal model, but the Caco-2 model had the least differentially expressed genes, which was surprising considering that this model is a cancerous cell line and consists only of epithelial cells. Interestingly, comparison of 492 intestine-specific genes showed that hiPSC-derived IEC layers have the highest expression of intestine-specific genes of all models. The hiPSCs-derived IEC layers and HIOs, and the Caco-2 cell layers showed a higher expression of genes related to immune function and signaling pathways suggesting that these models might be more sensitive in their responses to immune stimuli or toxicants. Furthermore, they also showed a lower expression of genes related to DNA replication and DNA repair pathways. Since the main focus of this study lies on transport of PFASs, the expression of intestinal transporters was evaluated in more detail. The hiPSC-derived IEC layers expressed substantial levels of relevant intestinal transporters at gene level. In fact, of the 37 evaluated genes 20 genes were significantly higher expressed in the hiPSC-derived IEC layers than in the EpiIntestinal model, whereas 8 genes had a significantly lower expression. In Caco-2 cell layers, there were 18 significantly higher and 9 lower expressed genes compared with the EpiIntestinal model. Comparison of the Caco-2 cell model with the hiPSC-derived IEC layers showed a generally lower differential expression of the transporter genes in the Caco-2 model. This suggests that the IEC model is likely well suited for transport studies, including studies on active transport. To validate this, the expression of transporters in the hiPSC-derived IEC layers should be confirmed, for instance using proteomics, immunohistochemistry or by measuring the activity of the transporters, which was out of the scope of this study.

In the present study, hiPSCs were differentiated into IEC layers for bidirectional transport studies. The transport data of the model compounds in the iPSC-derived IEC layers were consistent with the Biopharmaceuticals Classification System, showing higher transport for warfarin, propranolol and quinidine and lower transport for atenolol, enalapril and acebutolol, indicating that the hiPSC-derived IEC layers can clearly discriminate between high and low permeable compounds. When comparing the three different models, the order of transport of the low- and high-permeable compounds was largely comparable, but differences in the P_app_ values were seen. In general, the apparent permeability was higher in the hiPSC-derived IEC layers than in the other two models. Apart from the hydrophobicity and size of a compound, the intestinal apparent permeability of a compound is mainly determined by the barrier integrity, the cellular composition and the presence of uptake and efflux transporters (Markus et al. 2021; Vanuytsel et al. 2021; Franco et al. 2021) implicating that several factors or a combination of factors could be the cause of the observed differences. Caco-2 cell layers are known to generally have a high barrier integrity as indicated by high TEER values ranging from 300 to 2400 Ω cm^2^, which is dependent on clonal type and culture parameters (Markus et al. 2021). In contrast, the TEER values of the EpiIntestinal model have been reported to lie between 90 and 300 Ω cm^2^ (Markus et al. 2021). In the present study, only Caco-2 cell layers with TEER values > 400 Ω cm^2^ were used and the average TEER values of the hiPSC-derived IEC models was 330 Ω cm^2^ during the transport studies (data not shown). Another contributor to the apparent permeability is the cellular composition. While the Caco-2 model consists only of epithelial cells, the other two models consist of multiple cell types, but the exact ratio of the different cell types in the models is unknown and may differ between the models. For example, Caco-2 cell layers do not possess goblet cells, while hiPSC-derived IEC layers do, and the EpiIntestinal model has been described to contain mucus secreting granules which may point toward the presence of goblet cells as well. The presence of goblet cells, and as a result a mucus layer, also influences the apparent permeability of compounds. It has been reported that hydrophobic compounds have a reduced ability to migrate through mucus in comparison with hydrophilic compounds (Larhed et al. 1997). Additionally, the EpiIntestinal model contains a layer of fibroblasts that the other two models lack, which may also influence the apparent permeability. A last contributor to the apparent permeability is the presence of uptake and efflux transporters. The gene expression data on the expression of transporter genes (Fig. 5) showed that there is a distinct possibility that the presence of uptake and efflux transporters varies between the three models. This could influence the apparent permeability values for some of the compounds that have been described to involve active transport mechanisms (e.g., quinidine and acebutolol) (Terao et al. 1996; Ishida et al. 2006). Clearly, all three models have their own specific differences influencing the transport of chemicals which makes it hard to directly compare the intestinal models studied in the present study. The selection of the model compounds was based on the availability of transport data in literature. As can be expected, most transport data is available for the Caco-2 cell model. Comparing reported P_app_ values from literature using the Caco-2 cell model for the compounds used in this study (Supplementary Table 10) clearly illustrates that with the Caco-2 cell model large differences in *P*_app_ values have been reported. These variations in *P*_app_ values could be related to robustness of the model or to variation in study set-ups which would call for harmonization of study designs to reduce interlaboratory variation between *P*_app_ values. For the hiPSC-derived IEC layers, it is interesting to note that in the study by Kabeya et al. (2020), that first described the differentiation method to obtain hiPSC-derived IEC layers which was applied in this study, the transport data on enalapril and acebutolol were comparable to the data from this study (see Table 2). These initial findings are promising, but an extensive interlaboratory comparison on both models using harmonized protocols would be needed to conclude whether this method is more robust than the Caco-2 model. Nevertheless, the strength of these types of in vitro models might not lie in obtaining a quantitative *P*_app_ value, but in evaluation of relative transport properties of compounds, like was done in the experiment with PFASs in the present study.

Little is known about transport kinetics of PFASs and underlying mechanisms in the intestine. In the present study, the order of PFAS transport was highly similar between the three models, the *P*_app_ values in the hiPSC-derived IEC layers in A–B direction were in the following order PFHxS > PFOA > HFPO-DA > PFNA > PFOS. Currently, there are no quantitative PFASs absorption data from humans available, but based on animal studies it is estimated that some PFASs are rapidly and well absorbed (Gannon et al. 2011; Lupton et al. 2012). Several studies have examined active uptake and clearance mechanisms of PFASs in liver and kidney and have identified transporters that appear to be involved in active transport of PFASs. It is still unknown whether transporters are involved in the transport of PFAS in the intestine. Of the identified transporters so far, many are not present in the intestine, except for OATP2B1 and ASBT. These two transporters were shown to transport PFOS, PFHxS, PFOA and PFNA (Zhao et al. 2015, 2017; Kimura et al. 2017, 2020) and both transporters are expressed at mRNA levels in the tested models in this study. This may indeed suggest that these transporters played a role in the observed transport, but the efflux ratios suggest that there was no active transport of PFAS in the three models.

Currently, there are no data available about human exposure or the transport kinetics of HFPO-DA. In the present study, HFPO-DA transport was highest in the hiPSC-derived IEC model, while there was limited transport in the EpiIntestinal model and no transport in the Caco-2 model. Mass balance calculations showed that there were no indications that HFPO-DA was extensively metabolized, degraded or adsorbed to the plasticware in the Caco-2 samples in comparison with the other two models (Supplementary Fig. 3). A previous study (Kotlarz et al. 2020) measured HFPO-DA in serum of residents living near fluorochemical manufacturing facilities. HFPO-DA was present in water samples near the manufacturing facilities as well as other PFASs, such as PFHxS, PFOA, PFOS and PFNA. The latter were detected in serum of most participants at higher levels than the U.S. national levels for the 2015–2016 National Health and Nutrition Examination Survey, indicating exposure to PFASs which potentially included HFPO-DA, but HFPO-DA was not detected in serum. While this may indicate that HFPO-DA is not or limitedly absorbed, it might also be that serum HFPO-DA levels were below the limit of quantification or that HFPO-DA is quickly eliminated from the human body resulting in lower serum concentrations in humans compared to PFASs that are slowly eliminated. Clearly, more research is required to get better insights into the pharmacokinetics of HFPO-DA in the human body, for example by using isotope labeled HFPO-DA to allow easier tracing of HFPO-DA.

In summary, in the current study transport properties of the hiPSC-derived IEC model, the Caco-2 model and the EpiIntestinal model were evaluated. Our data showed similar functional properties as the conventional Caco-2 model and the EpiIntestinal model, with some differences in absolute measured values. In addition, our data indicated that the hiPSC-derived IEC model highly resembles human intestinal physiology, due to the presence of multiple intestinal cell types, and is therefore a promising novel in vitro model to study transport of chemicals across the intestinal barrier. Furthermore, the present study provides insight into the intestinal transport kinetics of PFASs, which was highest for PFHxS in all models and lowest for PFOS and HFPO-DA.

## Supplementary Information

Below is the link to the electronic supplementary material.

Supplementary file1 (DOCX 1275 KB)

## Data Availability

All datasets used and/or analyzed to support the findings of this study are available in this article or in the Supplementary Information. All the raw data supporting the results of this study are available from the corresponding author upon request.

## Abbreviations

HFPO-DA: Hexafluoropropylene oxide-dimer acid
hiPSCs: Human induced pluripotent stem cells
IECs: Intestinal epithelial cells
PFASs: Per- and polyfluoroalkyl substances
PFHxS: Perfluorohexane sulfonate
PFNA: Perfluorononanoic acid
PFOA: Perfluorooctanoic acid
PFOS: Perfluorooctane sulfonate
