# Supplementary material for: Transport of perfluoroalkyl substances across human induced pluripotent stem cell-derived intestinal epithelial cells in comparison with primary human intestinal epithelial cells and Caco-2 cells
Source: Arch Toxicol. 2024 Aug 31;98(11):3777–95. doi: 10.1007/s00204-024-03851-x (PMC11489206; doi:10.1007/s00204-024-03851-x)
Supplement: Supplementary file 1 — Supplementary file1 (DOCX 1275 KB) [file 204_2024_3851_MOESM1_ESM.docx]

**Supplementary data for:**

**Transport of perfluoroalkyl substances across human induced pluripotent stem cell-derived intestinal epithelial cells in comparison with primary human intestinal epithelial cells and Caco-2 cells**

Aafke W.F. Janssen^1*^, Loes P.M. Duivenvoorde^1^, Karsten Beekmann^1^, Nicole Pinckaers^1^, Bart van der Hee^2^, Annelies Noorlander^1^, Liz L. Leenders^1^, Jochem Louisse^1#^ Meike van der Zande^1^

^1^Wageningen Food Safety Research (WFSR), part of Wageningen University and Research, Akkermaalsbos 2, 6708 WB Wageningen, The Netherlands

^2^Animal Sciences Group, Wageningen University, De Elst 1, 6708 WD Wageningen, The Netherlands

^#^ current affiliation: European Food Safety Authority (EFSA), Parma, Italy

*** Corresponding author:**

Aafke Janssen PhD

Wageningen Food Safety Research

Akkermaalsbos 2

6708 WB Wageningen

The Netherlands

Phone: +31 317 483670

Email: aafke.janssen@wur.nl

**Materials and Methods**

***Chemicals***

**Supplementary Table 1. Suppliers, purities, catalog numbers and CAS numbers of chemicals tested in the present study**

| **Chemical** | **Full name** | **Supplier** | **Purity** | **Catalog number** | **CAS number** |
| --- | --- | --- | --- | --- | --- |
| Warfarin | - | Sigma-Aldrich | 100% | A2250-10G | 81-81-2 |
| Propranolol hydrochloride | - | Sigma-Aldrich | >99% | P0884-1G | 318-98-9 |
| Quinidine | - | Sigma-Aldrich | 89% | Q3625-5G | 56-54-2 |
| Atenolol | - | Sigma-Aldrich | 100% | A7655-1G | 29122-68-7 |
| Enalapril | - | Sigma-Aldrich | >99% | E6888-250MG | 76095-16-4 |
| Acebutolol | - | Sigma-Aldrich | 99% | A3669-1G | 34381-68-5 |
| PFOA | Perfluorooctanoic acid | Sigma-Aldrich | 95% | 171468-5G | 335-67-1 |
| PFNA | Perfluorononanoic acid | Sigma-Aldrich | 99% | 91977-50MG | 375-95-1 |
| PFHxS | Perfluorohexane sulfonate | Synquest laboratories | 95% | 6164-3-2T | 355-46-4 |
| PFOS | Perfluorooctane sulfonate | Synquest laboratories | 97% | 6164-3-08 | 1763-23-1 |
| HFPO-DA  (GenX) | Hexafluoropropylene oxide dimer acid | Synquest laboratories | 97% | 2121-3-13 | 13252-13-6 |

***hiPCS-derived IEC layer integrity***

To determine barrier integrity of the hiPSC-derived IEC layers, apical to basolateral translocation of lucifer yellow was measured. 500 µg/ml Lucifer yellow, in differentiation medium, containing EGF, Forskolin, 5-aza-2′-deoxycytidine, PD98059, A-83-01, was added to the apical compartment (400 µl/insert) and incubated at 37˚C for 3 hours. 50 µl medium was collected from the basolateral compartment at 30 min intervals. The fluorescence intensity was measured at 485 nm excitation and 530 nm emission wavelength using a microplate reader (Synergy HT, BioTek, Winooski, VT).

***RNA isolation and qPCR***

Total RNA was extracted from the hiPSCs-derived IEC layers using the RNeasy Mini Kit (Qiagen, Venlo, The Netherlands). Subsequently, 500 ng RNA was used to synthesize cDNA using the iScript cDNA synthesis kit (Bio-Rad Laboratories, Veenendaal, The Netherlands). Changes in gene expression were determined by real-time PCR on a CFX384 real-time PCR detection system (Bio-Rad Laboratories) using SensiMix (Bioline; GC Biotech, Alphen aan den Rijn, The Netherlands). The PCR conditions consisted of an initial denaturation at 95°C for 10 min, followed by 40 cycles of denaturation at 95°C for 10 s and annealing extension at 60°C for 15 s. The housekeeping gene RPL27 was used for normalization of gene expression. Primer sequences were taken from the Harvard PrimerBank and ordered from Eurogentec (Liège, Belgium). Sequences of the used primers are listed in **Table 1**.

To calculate the relative expression of the gene of interest, absolute quantification was performed using a standard curve. A six-point standard curve was derived from serial dilution of pooled cDNA samples. This standard curve was used to determine the amplification efficiency for each primer pair, which fell within the acceptable range between 90 and 110% (Rogers-Broadway and Karteris 2015). Gene expression analysis was performed using arbitrary starting quantities for the genes of interest and the housekeeping gene *RPL27* that were calculated by CFX Maestro Software (version 2.3) based on the standard curve. To normalize the gene expression data, the arbitrary starting quantity of the gene of interest was divided by the arbitrary starting quantity of *RPL27*. Finally, the normalized expression data were expressed relative to the phase with the highest expression of the gene of interest, which was set to one.

**Supplementary Table 2. Primer sequences used for qPCR**

| **Gene name** | **Primer sequence** |  |
| --- | --- | --- |
|  | **Forward** | **Reverse** |
| *RPL27* | ATCGCCAAGAGATCAAAGATAA | TCTGAAGACATCCTTATTGACG |
| *CDX2* | CCAGCGGCGGAACCTGTG | GTCTTTCGTCCTGGTTTTCAC |
| *IFABP* | CGCCCAAGGACAGACCTGAAT | TTCCAAGTGCTGTCAAACGCC |
| *VIL1* | CGGAAAGCACCCGTATGGAG | CGTCCACCACGCCTACATAG |
| *SI* | TCCAGCTACTACTCGTGTGAC | CCCTCTGTTGGGAATTGTTCTG |
| *LGR5* | GGAAATCATGCCTTACAGAGC | CACTCCAAATGCACAGCACTG |
| *LYZ* | CCCTGGTCAGCCTAGCACTC | CCTTGCCCTGGACCGTAACA |
| *MUC2* | AGAAGGCACCGTATATGACGAC | CAGCGTTACAGACACACTGCTC |
| *CHGA* | TCCGACACACTTTCCAAGCC | TTCTGCTGATGTGCCCTCTC |
| *ZO1* | CAACATACAGTGACGCTTCACA | CACTATTGACGTTTCCCCACTC |
| *OCCLUDIN* | ACAAGCGGTTTTATCCAGAGTC | GTCATCCACAGGCGAAGTTAAT |
| *E-CADHERIN* | ATTTTTCCCTCGACACCCGAT | TCCCAGGCGTAGACCAAGA |

***Immunofluorescence staining***

**Supplementary Table 3. Antibodies used for IEC layer characterization**

|  |
| --- |

| \| **Antibody** \| **Target** \| **Supplier** \| **Catalog number** \| **Antibody ID** \| \| --- \| --- \| --- \| --- \| --- \| \| UEA1 \| Surface lectins \| ThermoFisher \| L32476 \| - \| \| GLP1 \| EEC L cells \| Mybiosource \| MBS2090479 \| - \| \| LGR5 \| Stem cells \| Invitrogen \| PA5-23000 \| AB_11153755 \| \| TFF3 \| Goblet cells \| Mybiosource \| MBS2057630 \| - \| \| VIL1 \| Enterocytes \| Abcam \| AB3304 \| GR217362-1 \| \| ZO1 \| Tight junction \| ThermoFisher \| 339194 \| UA276783 \| \| Alexa Fluor 488 (goat)  Alexa Fluor 488 (donkey) \| Rabbit  Mouse \| ThermoFisher  Abcam \| A32731  AB150105 \| - \| \| Alexa Fluor 555 (goat) \| Mouse \| ThermoFisher \| A21422 \| - \| \| Alexa Fluor 647 (goat) \| Rabbit \| ThermoFisher \| A21244 \| - \| |
| --- | --- | --- | --- | --- | --- | --- | --- | --- | --- | --- | --- | --- | --- | --- | --- | --- | --- | --- | --- | --- | --- | --- | --- | --- | --- | --- | --- | --- | --- | --- | --- | --- | --- | --- | --- | --- | --- | --- | --- | --- | --- | --- | --- | --- | --- | --- | --- | --- | --- | --- |
| UEA1; Ulex europaeus agglutinin 1, GLP1; Glucagon-like peptide 1, EEC; enteroendocrine, LGR5; leucine-rich repeat-containing G protein-coupled receptor 5, TFF3; Trefoil factor 3, VIL1; Villin, ZO1; zonula occludens 1. |

***RNA Library Preparations and RNA seq***

Approximately 1 microgram total RNA was used for RNA library preparations using a TruSeq Stranded mRNA Sample Prep kit (Illumina). After polyA based mRNA selection, RNA was further processed including subsequent fragmentation, first and second strand cDNA synthesis, adapter ligation and final library amplification resulting in RNA seq libraries including unique dual indexes, all following manufacturer’s protocol. Final libraries were eluted in 30 µl elution buffer followed by library quality assessment using a Fragment Analyzer (Agilent Technologies) and quantified by Qubit fluorescence measurements (Life Technologies).

Prepared libraries were pooled in an equimolar manner and combined with other indexed libraries for sequencing on an Illumina NovaSeq 6000 system. Final sequencing was done using an S2 and S4 type flow cell, both with an XP loading workflow and settings specific for 2x150 nt paired end reads plus dual indexes reads. All steps for sequencing were carried out according to manufacturer’s protocol. Demultiplexing of reads per sample by corresponding indexes was done using bcl2fastq v2.20.0.422 (Illumina Inc, San Diego, CA, USA).

***Processing of RNA seq reads***

RNA seq reads were used to quantify transcript abundances. To this end, the tool Cutadapt (version 1.16) (Martin 2011) was used to trim adapters from the reads and HISAT2 (version 2.2.1) (Kim et al. 2019) was used to map the reads to the GRCh38.p13 human genome assembly-based transcriptome sequences as annotated by the GENCODE consortium (release 40). HISAT2 output was converted and sorted by chromosomal position using Samtools (version 1.9) (Danecek et al. 2021). RSeQC (v3.0.1) (Wang et al. 2012) and PRINSEQ (v0.20.4) (Schmieder and Edwards 2011) were used for quality control. HTSeq (version 0.11.2) (Anders et al. 2015) was used to count reads in transcripts using gene-level quantification. Separately, DESeq2 (v1.34.0) (Love et al. 2014) normalized the count data from HTSeq using a median of ratios based method, which was used for preparation of heatmaps and performing gene set enrichment analysis. Differential gene expression was determined using the package limma (version 3.50.3) (Ritchie et al. 2015) utilizing the obtained scaled gene-level counts. Briefly, before statistical analyses, nonspecific filtering of the count table was performed to increase detection power, based on the requirement that a gene should have an expression level greater than around 10 counts in at least one triplicate. Differences in library size were adjusted by the trimmed mean of M-values normalization method, implemented in the package edgeR (version 3.36.0) (Robinson et al. 2009; McCarthy et al. 2012; Chen et al. 2016). Counts were transformed to log2 (counts per million; cpm) values and associated precision weights, and entered into the limma analysis pipeline. Differentially expressed genes were identified by using generalized linear models that incorporate empirical Bayes methods.

***Transport studies***

Transport studies were performed with Caco-2 cells, hiPSC-derived IECs and EpiIntestinal microtissues. To that end, Caco-2 cell layers were differentiated for 21 days and hiPSCs were differentiated into IEC layers for 26 days. EpiIntestinal microtissues were subjected to transport studies one day after arrival.

Prior to exposure to the transport study compounds, the cells were washed with transport medium (HBSS; Sigma). For apical to basolateral transport in the EpiIntestinal microtissues, 0.5 mL HBSS was added to the basolateral compartment and 0.2 mL HBSS containing either 10 µM Warfarin, 10 µM Atenolol or a mixture of PFASs containing 1 µM of each PFAS, was added to the apical compartment. 100 µL aliquots were collected from the basolateral compartment and replaced with the same volume HBSS at settled time points (0, 15, 30 and 60 min). For basolateral to apical transport in the EpiIntestinal microtissues, 0.2 mL HBSS was added to the apical compartment and 0.5 mL HBSS containing a mixture of PFASs containing 1 µM of each PFAS, was added to the basolateral compartment. 100 µL aliquots were collected from the apical compartment and replaced with the same volume of HBSS at settled time points (0, 15, 30 and 60 min). For apical to basolateral transport across the Caco-2 cell layers and hiPSC-derived IEC layers, 1.2 mL HBSS was added to the basolateral compartment and 0.4 mL HBSS containing either model compounds (10 µM) or a mixture of PFASs containing 1 µM of each PFAS, was added to the apical compartment. 100 µL aliquots were collected from the basolateral compartment and replaced with the same volume of HBSS at settled time points (0, 7.5, 15, 30 and 60 min for the model compounds with both models and the PFASs with the hiPSC-derived IEC layers and 0, 15, 30 and 60 min for PFASs with the Caco-2 cell layers). For basolateral to apical transport, 0.4 mL HBSS was added to the apical compartment and 1.2 mL HBSS containing 10 µM of the model compounds or a mixture of PFASs containing 1 µM of each PFAS, was added to the basolateral compartment. 100 µL aliquots were collected from the apical compartment and replaced with the same volume of HBSS at settled time points (0, 7.5, 15, 30 and 60 min for the model compounds with both models and the PFASs with the hiPSC-derived IEC layers and 0, 15, 30 and 60 min for PFASs with the Caco-2 cell layers). After 60 minutes, all liquid was collected from the apical and basolateral compartment and the cells were lysed in 200 µL RIPA buffer (Thermo Fisher Scientific), and chemicals were quantified in the different compartments (apical, basolateral and cells) in order to calculate the mass balance.

The apparent permeability coefficient (P_app_, cm/s) was calculated using the following equation:

$$P_{app}=\frac{dQ}{dt} \times\frac{1}{A \times C{}_{0}}$$

where *A* is the surface area (cm^2^), *dQ* is the amount of the compound transported (nmol) over the respective time interval *dt* (s) and *C_0_* is the initial concentration in the donor compartment (µM).

In the bidirectional permeability studies, the efflux ratio (ER) was calculated according to the following equation:

$$ER= \frac{P{}_{app}(BA)}{P_{app}(AB)}$$

The mass balance (recovery) of the PFASs was defined as the sum of the test compound remaining in the donor compartment at the end of the transport study along with the quantities found in the cell lysate and recovered from the receiver compartment, divided by the initial donor amount as quantified by LC/MS. The recovery was calculated using the following equation:

$$Recovery \left( \% \right)= \frac{C_{DEND}\cdot V_{D} +C_{L}\cdot V_{L}+C_{R}\cdot V_{R}}{C_{DSTART}\cdot V_{D}} x100\%$$

where *C_DEND_* and *C_DSTART_* are the concentration on the donor compartment at the end and start of the experiment, *C_L_* is the concentration in the cell lysates and *C_R_* the concentration in the receiver compartment at the end of the study. *V_D_*, *V_L_* and *V_R_* are the respective volumes.

***Sample quantification (LC/MS-MS) model compounds***

**Supplementary Table 4. Compounds and MRM parameters used in this study**

| **Compound** | **Ion mode** | **Mass charge (m/z) transitions** | **Cone voltage (V)** | **Collision energy (V)** |
| --- | --- | --- | --- | --- |
| Acebutolol | Positive | 337.21 > 116.10  337.21 > 319.20 | 30 | 25  15 |
| Atenolol | Positive | 267.00 > 56.41  267.00 > 145.00 | 20 | 35  25 |
| Enalapril | Positive | 377.21 > 117.07  377.21 > 234.15 | 30 | 40  20 |
| Propranolol | Positive | 260.00 > 116.00 | 30 | 30 |
| Quinidine | Positive | 325.19 > 117.05  325.19 > 160.07  325.19 > 172.02 | 30 | 25  25  25 |
| Warfarin | positive | 309.2 > 162.9  309.2 > 250.9 | 20 | 15  20 |

|  |
| --- |

***Sample quantification (LC/MS-MS) PFASs***

**Supplementary Table 5. MRM transitions of the PFASs tested in the present study as used in the Shimadzu system**

| **Name** | **Q1 Mass Da** | **Q3 Mass Da** | **Dwell (msec)** | **DP** | **EP** | **CE** | **CXP** |
| --- | --- | --- | --- | --- | --- | --- | --- |
| ^13^C_4_-PFOA | 416.9 | 371.9 | 4.0 | -40 | -15 | -24 | -19 |
| ^13^C_5_-PFNA | 468.0 | 423.0 | 4.0 | -75 | -10 | -16 | -27 |
| ^18^O_2_-PFHxS | 403.0 | 84.0 | 4.0 | -30 | -10 | -40 | -8 |
| ^13^C_4_-PFOS | 502.9 | 99.0 | 4.0 | -80 | -5 | -34 | -7 |
| ^13^C_3_-HFPO-DA | 332.1 | 169.1 | 4.0 | -75 | -10 | -14 | -21 |
| PFOA | 412.9 | 369.1 | 4.0 | -40 | -10 | -14 | -11 |
| PFOA | 412.9 | 169.0 | 4.0 | -40 | -15 | -24 | -19 |
| PFNA | 462.9 | 419.1 | 4.0 | -75 | -10 | -16 | -27 |
| PFNA | 462.9 | 169.0 | 4.0 | -75 | -10 | -26 | -11 |
| PFHxS | 398.9 | 80.0 | 4.0 | -110 | -10 | -104 | -17 |
| PFHxS | 398.9 | 98.9 | 4.0 | -110 | -10 | -42 | -15 |
| PFOS | 498.9 | 99.0 | 4.0 | -80 | -5 | -94 | -7 |
| PFOS | 498.9 | 80.0 | 4.0 | -80 | -5 | -100 | -11 |
| HFPO-DA | 328.9 | 169.1 | 4.0 | -5 | -10 | -18 | -13 |
| HFPO-DA | 328.9 | 285.0 | 4.0 | -5 | -10 | -6 | -19 |

**Supplementary Table 6. MRM transitions of the PFASs tested in the present study as used in the Sciex system**

| **Name** | **Q1 Mass Da** | **Q3 Mass Da** | **Dwell (msec)** | **EP** | **CE** | **CXP** |
| --- | --- | --- | --- | --- | --- | --- |
| ^13^C_4_-PFOA | 417.0 | 372.0 | 9.30 | -10 | -15 | -17 |
| ^13^C_5_-PFNA | 468.0 | 423.0 | 10.1 | -10 | -16 | -29 |
| ^18^O_2_-PFHxS | 403.0 | 83.9 | 9.64 | -10 | -74 | -9 |
| ^13^C_4_-PFOS | 503.0 | 98.9 | 7.39 | -10 | -95 | -11 |
| ^13^C_3_-HFPO-DA | 287.0 | 169.0 | 11.5 | -10 | -17 | -5 |
| PFOA | 413.0 | 169.0 | 9.30 | -10 | -26 | -9 |
| PFOA | 413.0 | 369.0 | 9.30 | -10 | -15 | -7 |
| PFNA | 463.0 | 419.0 | 10.1 | -10 | -17 | -8 |
| PFNA | 463.0 | 219.0 | 10.1 | -10 | -24 | -11 |
| PFHxS | 399.0 | 79.9 | 9.64 | -10 | -88 | -9 |
| PFHxS | 399.0 | 98.9 | 9.64 | -10 | -75 | -11 |
| PFOS | 499.0 | 79.9 | 7.39 | -10 | -104 | -9 |
| PFOS | 499.0 | 98.9 | 7.39 | -10 | -95 | -11 |
| HFPO-DA | 285.0 | 169.0 | 11.5 | -10 | -11 | -9 |
| HFPO-DA | 285.0 | 185.0 | 11.5 | -10 | -23 | -11 |

**Results**

**
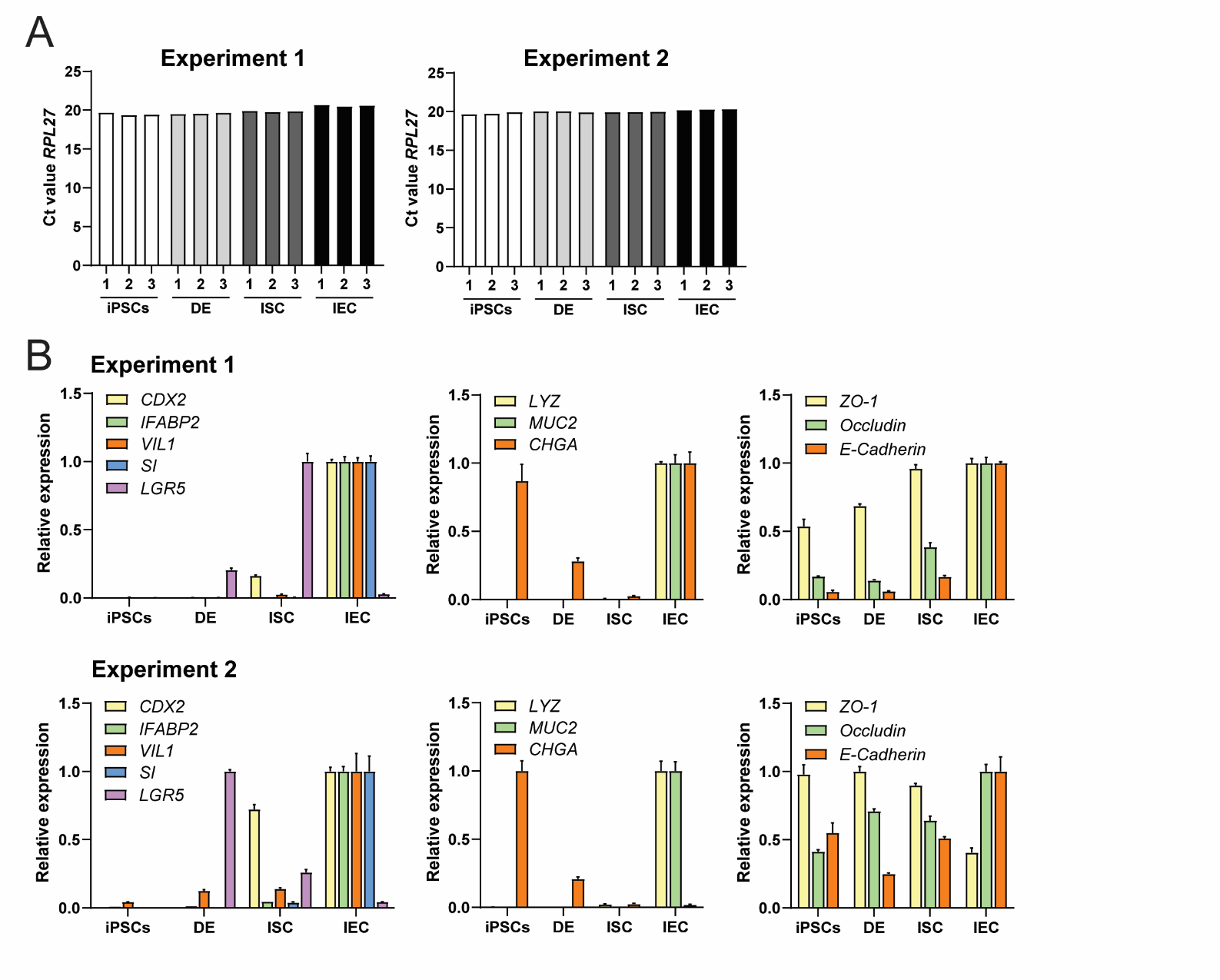
*Differentiation and characterization of hiPSC-derived intestinal epithelial cell layers***

**Supplementary Figure 1. Differentiation of hiPSCs into intestinal epithelial cell (IEC) layers from two independent experiments.** **A.** Ct values of the housekeeping gene *RPL27* throughout all phases of differentiation. Each independent experiment contained triplicates per differentiation phase. **B.** Relative expression of intestinal differentiation markers, stem cell marker LGR5, tight junction and adherens junction proteins during the course of the differentiation from hiPSCs up to IEC layers. The highest expression level of each gene was set at one. Data are presented as mean values ± SEM from triplicate wells.

**Supplementary Table 7. Ct values of intestinal markers in hiPSC-derived intestinal epithelial cell layers (of triplicate wells)**

| Gene | Mean ct value ± SEM |
| --- | --- |
| *CDX2* | 24.76 ± 0.11 |
| *IFABP2* | 29.21 ± 0.24 |
| *VIL1* | 23.37 ± 0.39 |
| *SI* | 28.90 ± 0.13 |
| *LGR5* | 31.36 ± 0.41 |
| *LYZ* | 26.28 ± 0.34 |
| *MUC2* | 23.91 ± 0.46 |
| *CHGA* | 29.87 ± 1.31 |
| *ZO-1* | 25.20 ± 0.17 |
| *Occludin* | 24.31 ± 0.07 |
| *E-Cadherin* | 22.84 ± 0.33 |

Data is obtained from 2 independent experiment with triplicate wells.


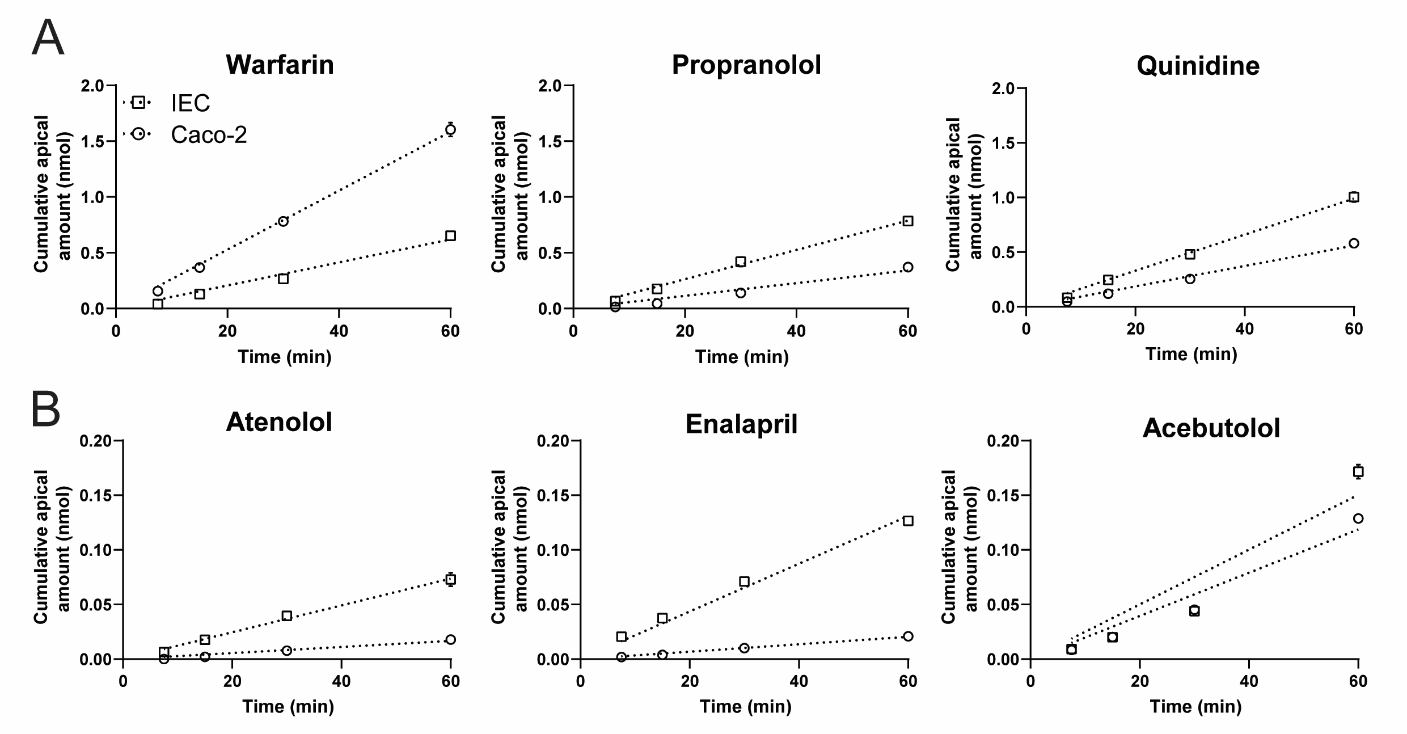
***Transport of model compounds***

**Supplementary Figure 2. Model compound excretion (B-A direction) in the hiPSC-derived IEC layers and Caco-2 layers.** Cumulative amount of (**A**) high and (**B**) low permeable compounds in the apical compartment during the course of the apical compartment at settled time points and model compounds were quantified using LC/MS-MS.

**Supplementary Table 8. Comparison of model compound apparent permeability (P_app_) values of B-A transport in the hiPSC-derived IEC and Caco-2 models**

|  | IEC | Caco-2 |  |
| --- | --- | --- | --- |
|  | Average P_app_  B – A  (x10^-6^ cm/s) | Average P_app_  B – A  (x10^-6^ cm/s) | BCS Class^1^ |
| Warfarin | 19.16 ± 0.30^a^ | 24.52 ± 0.94^b^ | 1 |
| Propranolol | 17.93 ± 0.93^a^ | 8.13 ± 0.12^b^ | 1 |
| Quinidine | 25.46 ± 1.17^a^ | 12.90 ± 0.47^b^ | 2 |
| Atenolol | 1.76 ± 0.15^a^ | 0.32 ± 0.04^b^ | 3 |
| Enalapril | 3.68 ± 0.10^a^ | 0.43 ± 0.05^b^ | 3 |
| Acebutolol | 7.17 ± 0.27^a^ | 3.09 ± 0.07^b^ | 3 |

Different letters indicate statistically significant differences at p < 0.05.

^1^ Data obtained from Ayehunie et al. (2005)

**
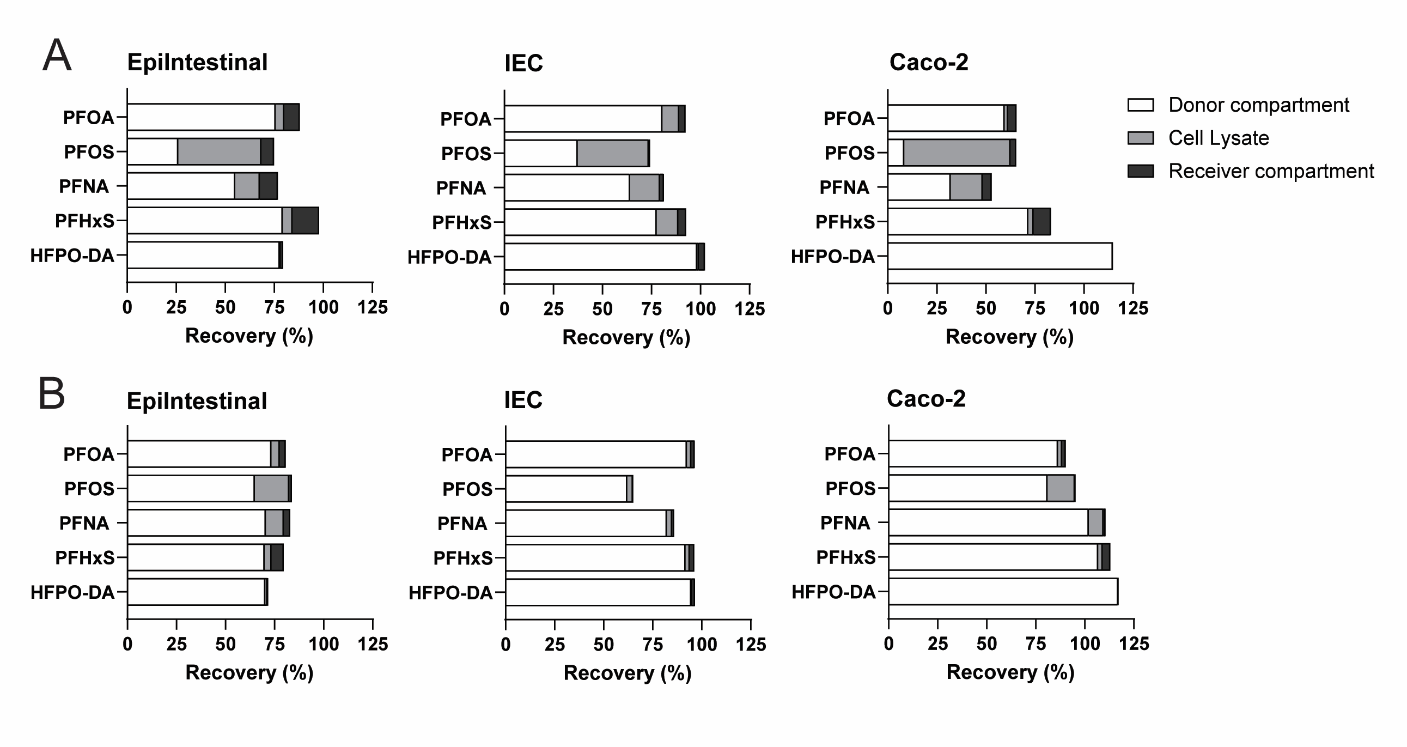
*Transport of PFASs***

**Supplementary Figure 3**. **Mass balances determined for the PFAS transport experiments.** **A.** In apical to basolateral direction. **B.** In basolateral to apical direction.

**
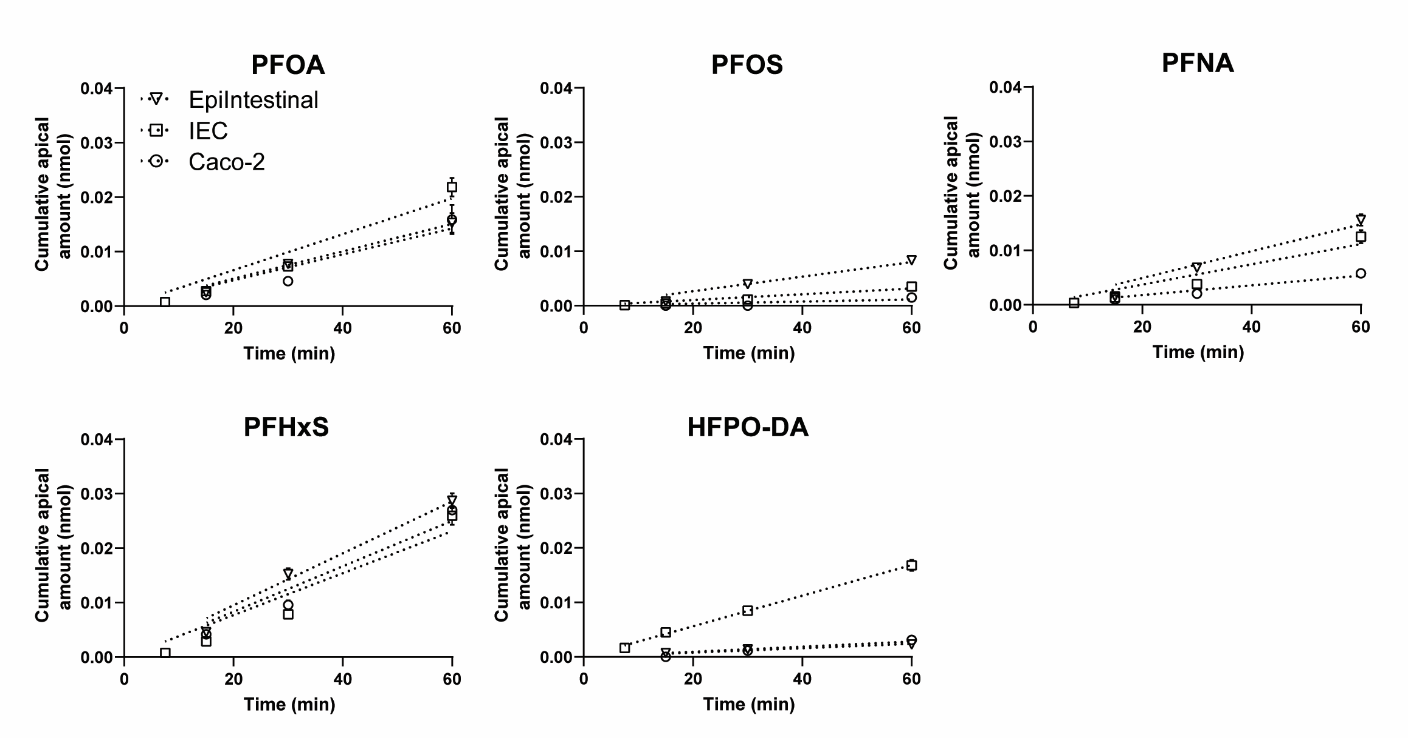
Supplementary Figure 4. PFAS excretion (B-A direction) in the EpiIntestinal model, hiPSC-derived IEC layers and Caco-2 layers. A.** Cumulative amount of PFOA, PFOS, PFNA, PFHxS and HFPO-DA in the apical compartment during the course of the transport study upon basolateral exposure to a mixture containing 1µM of each PFAS at t=0. Samples were taken from the apical compartment at settled time points and PFASs were quantified using LC/MS-MS. Data are mean values ± SEM from triplicate wells.

**Supplementary Table 9. Apparent permeability (P_app_) values of B-A transport of PFASs in the EpiIntestinal, hiPSC-derived IEC and Caco-2 models**

|  | EpiIntestinal | IEC | Caco-2 |
| --- | --- | --- | --- |
|  | Average P_app_  B - A  (x10^-6^ cm/s) | Average P_app_  B - A  (x10^-6^ cm/s) | Average P_app_  B - A  (x10^-6^ cm/s) |
| PFOA | 6.99 ± 0.82^a^ | 5.30 ± 0.42^a^ | 5.75 ± 0.97^a^ |
| PFOS | 3.31 ± 0.22^a^ | 0.82 ± 0.17^b^ | 1.06 ± 011^b^ |
| PFNA | 7.51 ± 0.50^a^ | 2.88 ± 0.28^b^ | 3.14 ± 0.33^b^ |
| PFHxS | 14.11 ± 0.67^a^ | 6.71 ± 0.43^b^ | 11.64 ± 0.20^c^ |
| HFPO-DA | 1.04 ± 0.09^a^ | 4.49 ± 0.27^b^ | 1.23 ± 0.25^a^ |

Different letters indicate statistically significant differences at p < 0.05 per compound.

**Discussion**

|  | Average P_app_ A - B  (x10^-6^ cm/s) | Reference |
| --- | --- | --- |
| Warfarin | 28.6 | Ayehunie *et al.* 2018 |
|  | 38.3 | Artursson *et al.* 1991 |
|  | 21.1 | Yazdanian *et al.* 1998 |
|  | 22.7 | Gschwind *et al*. 2013 |
| Propranolol | 12.9 | Ayehunie *et al.* 2018 |
|  | 41.9 | Artursson *et al.* 1991 |
|  | 35.3 | Walgren *et al.* 1999 |
|  | 21.8 | Yazdanian *et al.* 1998 |
|  | 34.4 | Grès *et al.* 1998 |
|  | 0.12 | Nožinić *et al*. 2010 |
|  | 36.13 | Logoyda *et al.* 2018 |
| Quinidine | 3.4 | Ayehunie *et al.* 2018 |
|  | 31.2 | Furubayashi *et al.* 2020 |
|  | 40.2 | Hellinger *et al.* 2012 |
|  | 20.4 | Yee 1997 |
|  | 20.1 | Logoyda *et al.* 2018 |
| Atenolol | 0.3 | Ayehunie *et al.* 2018 |
|  | 0.2 | Artursson *et al.* 1991 |
|  | 0.53 | Yazdanian *et al.* 1998 |
|  | 0.8 | Furubayashi *et al.* 2020 |
|  | 1.16 | Grès *et al.* 1998 |
|  | 0.83 | Hellinger *et al*. 2012 |
|  | 1.77 | Logoyda *et al.* 2018 |
| Enalapril | 0.2 | Ayehunie *et al.* 2018 |
|  | 0.19 | Kabeya *et al*. 2020 |
|  | 0.62 | Grès *et al.* 1998 |
|  | 1.31 | Logoyda *et al.* 2018 |
| Acebutolol | 0.40 | Ayehunie *et al.* 2018 |
|  | 0.51 | Yazdanian *et al.* 1998 |
|  | 0.35 | Kabeya *et al*. 2020 |
|  | 0.51 | Liang *et al.* 2000 |

**Supplementary Table 10. Comparison of P_app_ values in Caco-2 model for model compounds used in this study.**

**References**

Anders S, Pyl PT, Huber W (2015) HTSeq-A Python framework to work with high-throughput sequencing data. Bioinformatics 31:166–169. https://doi.org/10.1093/bioinformatics/btu638

Chen Y, Lun ATL, Smyth GK (2016) From reads to genes to pathways: Differential expression analysis of RNA-Seq experiments using Rsubread and the edgeR quasi-likelihood pipeline. F1000Res 5:1–51. https://doi.org/10.12688/F1000RESEARCH.8987.2

Danecek P, Bonfield JK, Liddle J, et al (2021) Twelve years of SAMtools and BCFtools. Gigascience 10:1–4. https://doi.org/10.1093/gigascience/giab008

Kim D, Paggi JM, Park C, et al (2019) Graph-based genome alignment and genotyping with HISAT2 and HISAT-genotype. Nat Biotechnol 37:907–915. https://doi.org/10.1038/s41587-019-0201-4

Love MI, Huber W, Anders S (2014) Moderated estimation of fold change and dispersion for RNA-seq data with DESeq2. Genome Biol 15:. https://doi.org/10.1186/s13059-014-0550-8

Martin M (2011) Cutadapt removes adapter sequences from high-throughput sequencing reads. EMBnet J 17:10–12

McCarthy DJ, Chen Y, Smyth GK (2012) Differential expression analysis of multifactor RNA-Seq experiments with respect to biological variation. Nucleic Acids Res 40:4288–4297. https://doi.org/10.1093/nar/gks042

Ritchie ME, Phipson B, Wu D, et al (2015) Limma powers differential expression analyses for RNA-sequencing and microarray studies. Nucleic Acids Res 43:e47. https://doi.org/10.1093/nar/gkv007

Robinson MD, McCarthy DJ, Smyth GK (2009) edgeR: A Bioconductor package for differential expression analysis of digital gene expression data. Bioinformatics 26:139–140. https://doi.org/10.1093/bioinformatics/btp616

Rogers-Broadway KR, Karteris E (2015) Amplification efficiency and thermal stability of qPCR instrumentation: Current landscape and future perspectives. Exp Ther Med 10:1261–1264. https://doi.org/10.3892/etm.2015.2712

Schmieder R, Edwards R (2011) Quality control and preprocessing of metagenomic datasets. Bioinformatics 27:863–864. https://doi.org/10.1093/bioinformatics/btr026

Wang L, Wang S, Li W (2012) RSeQC: Quality control of RNA-seq experiments. Bioinformatics 28:2184–2185. https://doi.org/10.1093/bioinformatics/bts356
